# Supplementary material for: Visible Light-Induced Cascade Sulfonylation/Cyclization to Produce Quinoline-2,4-Diones under Metal-Free Conditions
Source: Molecules. 2023 Mar 31;28(7):3137. doi: 10.3390/molecules28073137 (PMC10095780; doi:10.3390/molecules28073137)
Supplement: Supplementary file 1 [file molecules-28-03137-s001.zip › molecules-2314669-supplementary.pdf]

Supporting Information

Visible Light-Induced Cascade Sulfonylation/Cyclization to Produce Quinoline-2,4-Diones under Metal-Free Conditions

Yan Zhang [1,2], Ge Qiu [2,*], Fei Liu [2], Dongyang Zhao [2], Miao Tian [2] and Kai Sun [2,*]

[1] School of Chemistry and Chemical Engineering, Henan Normal University, Xinxiang 453007, China
[2] College of Chemistry and Chemical Engineering, Yantai University, Yantai 264005, China
* Correspondence: qiuge@zzu.edu.cn (G.Q.); sunk468@nenu.edu.cn (K.S.)

Table of Contents

I. Control Experiments 2

II. NMR Spectra of Products.. 4

I. Control Experiments

1.1 Control experiment in the presence of TEMPO

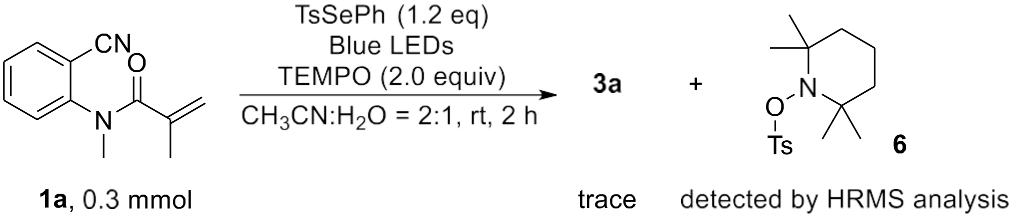

To a reaction tube, acrylamide **1a** (0.3 mmol), TsSePh (1.2 equiv, 0.36 mmol), the radical scavenger TEMPO (2.0 equiv, 0.6 mmol) were mixed in CH_3CN/H_2O (2:1, 2 mL). Irradiated for 2 h and monitored by TLC, we successfully detected the desired **6** by HRMS analysis.

HRMS (ESI) calcd for $C_{16}H_{26}NO_3S$ $[M+H]^+$: 312.1268, found: 312.1487.

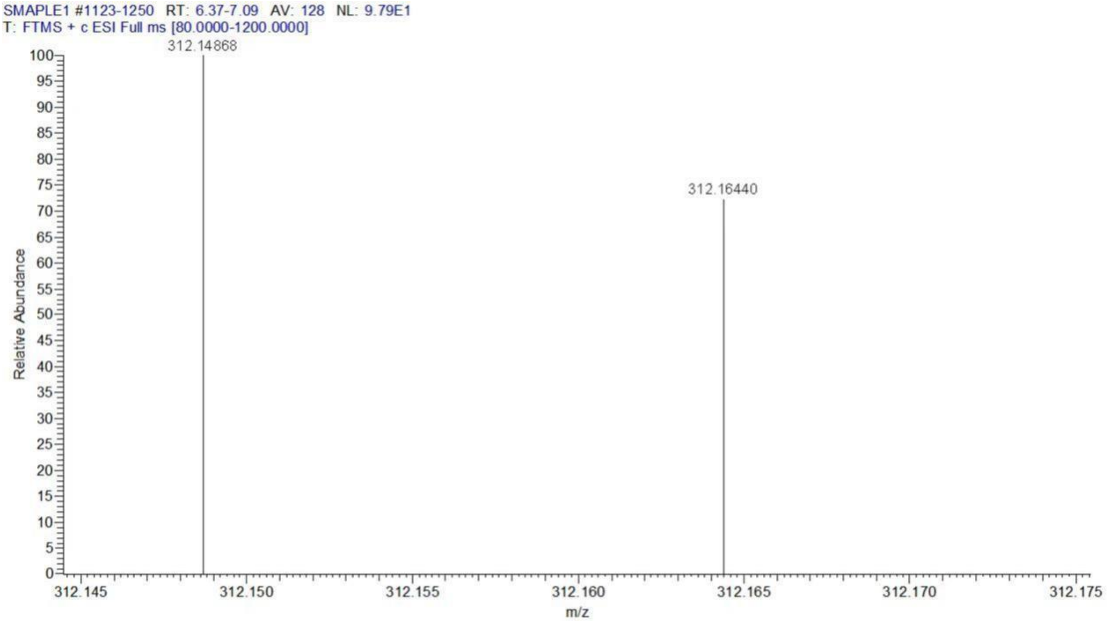

1.2 Control experiment in the presence of BHT

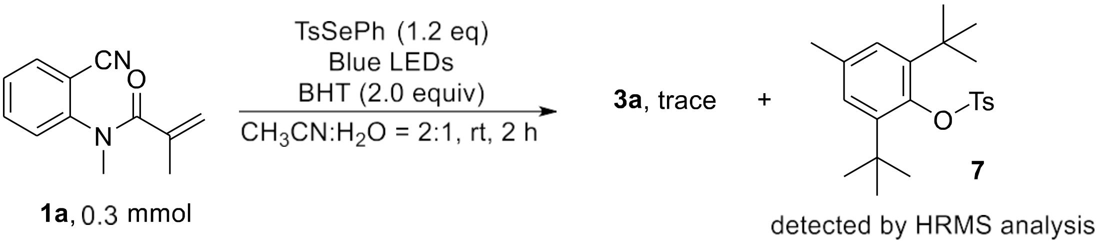

To a reaction tube, acrylamide **1a** (0.3 mmol), TsSePh (1.2 equiv, 0.36 mmol), the radical scavenger BHT (2.0 equiv, 0.6 mmol) were mixed in CH_3CN/H_2O (2:1, 2 mL). Irradiated for 2 h and monitored by TLC, we successfully detected the desired **7** by HRMS analysis.

HRMS (ESI) calcd for $C_{22}H_{31}O_3S$ $[M+H]^+$: 375.1988, found: 375.1985.

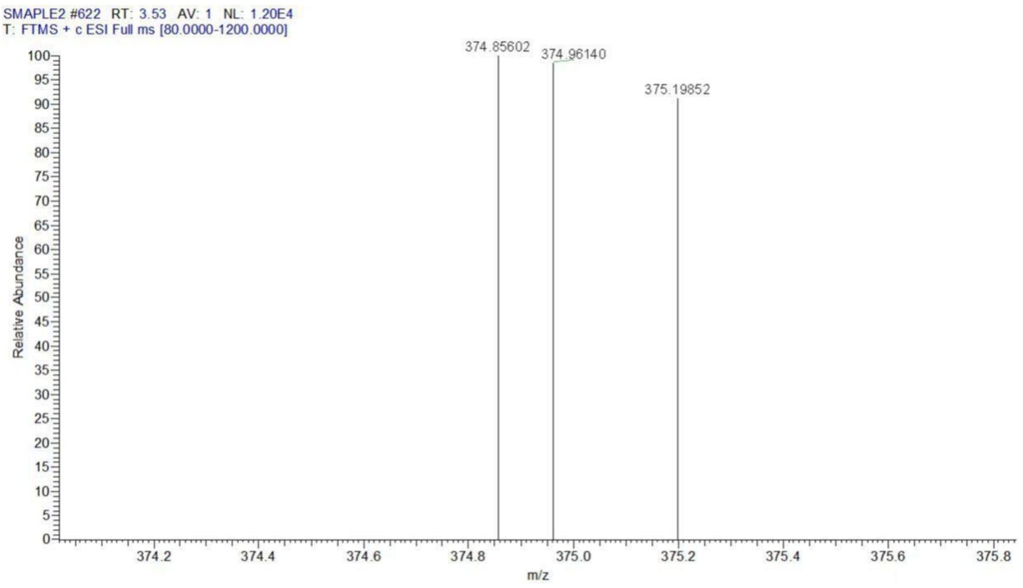

1.3 Control experiment in the presence of 1,1-diphenylethene

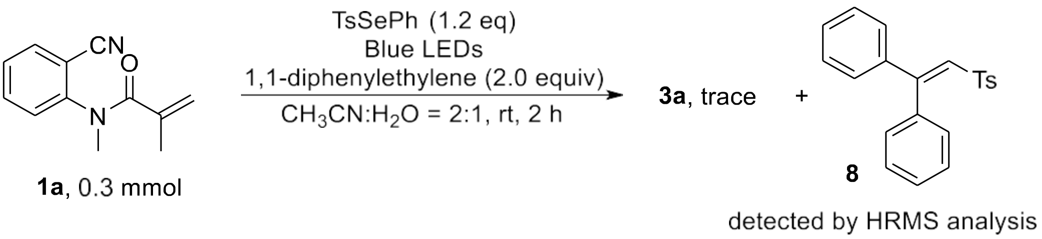

To a reaction tube, acrylamide **1a** (0.3 mmol), TsSePh (1.2 equiv, 0.36 mmol), the radical scavenger 1,1-diphenylethylene (2.0 equiv, 0.6 mmol) were mixed in CH_3CN/H_2O (2:1, 2 mL). Irradiated for 2 h and monitored by TLC, we successfully detected the desired **8** by HRMS analysis.

HRMS (ESI) calcd for $C_{21}H_{19}O_2S$ $[M+H]^+$: 335.1100, found: 335.1092.

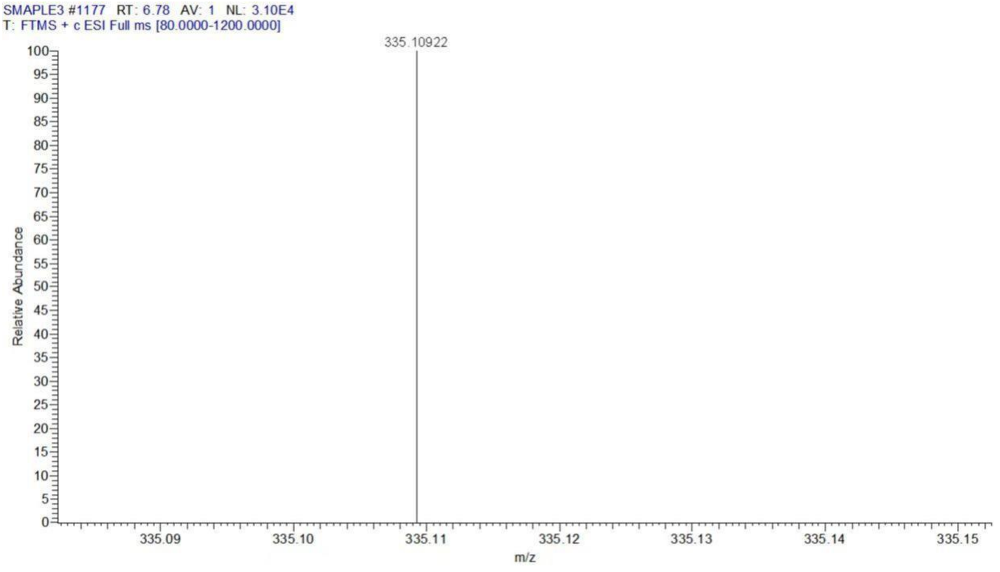

II. NMR Spectra of Products

Compound 3a

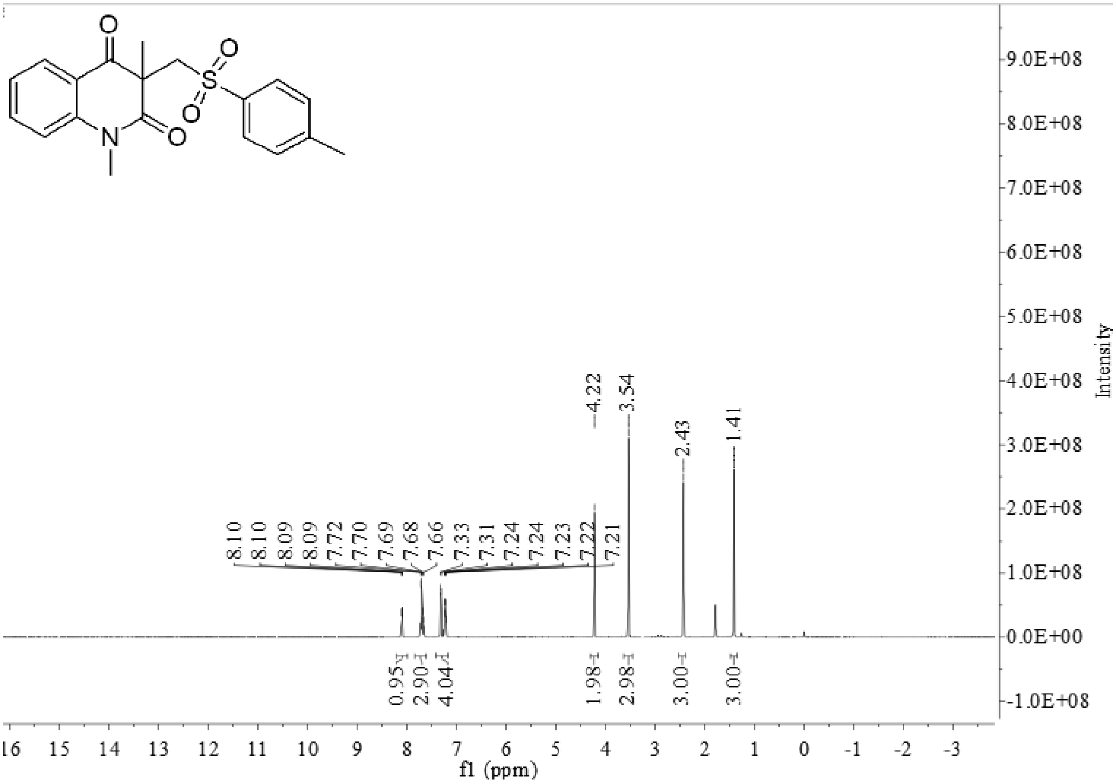

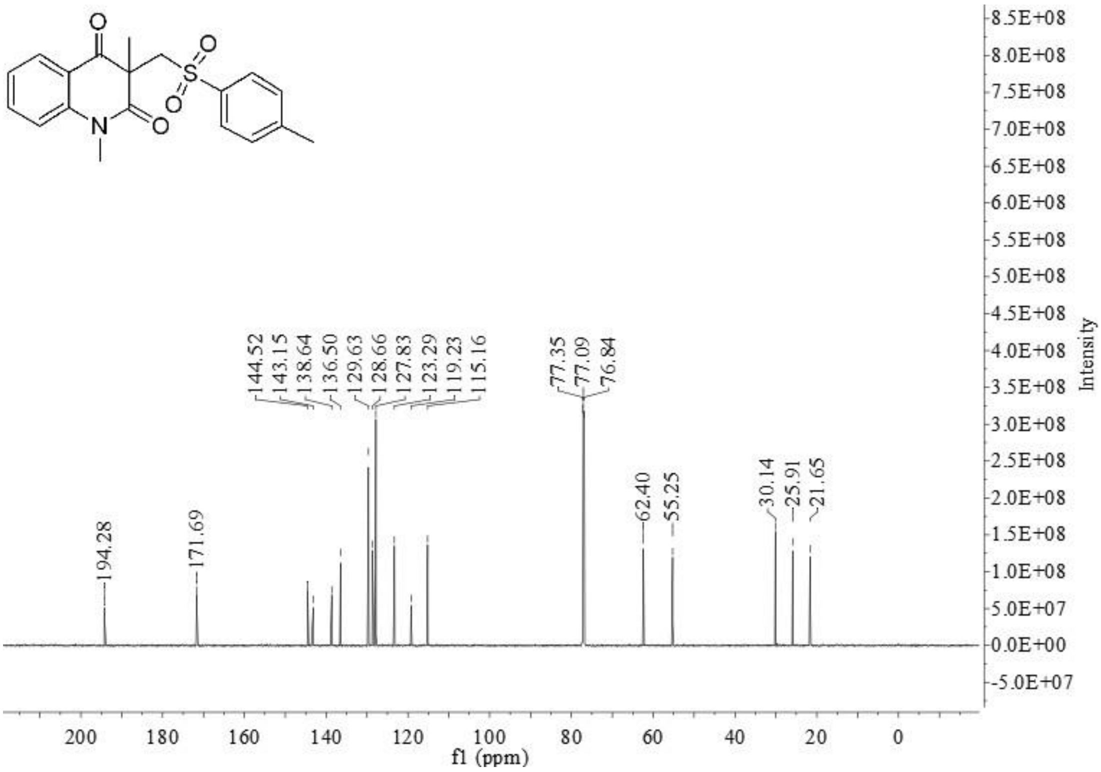

Compound 3b

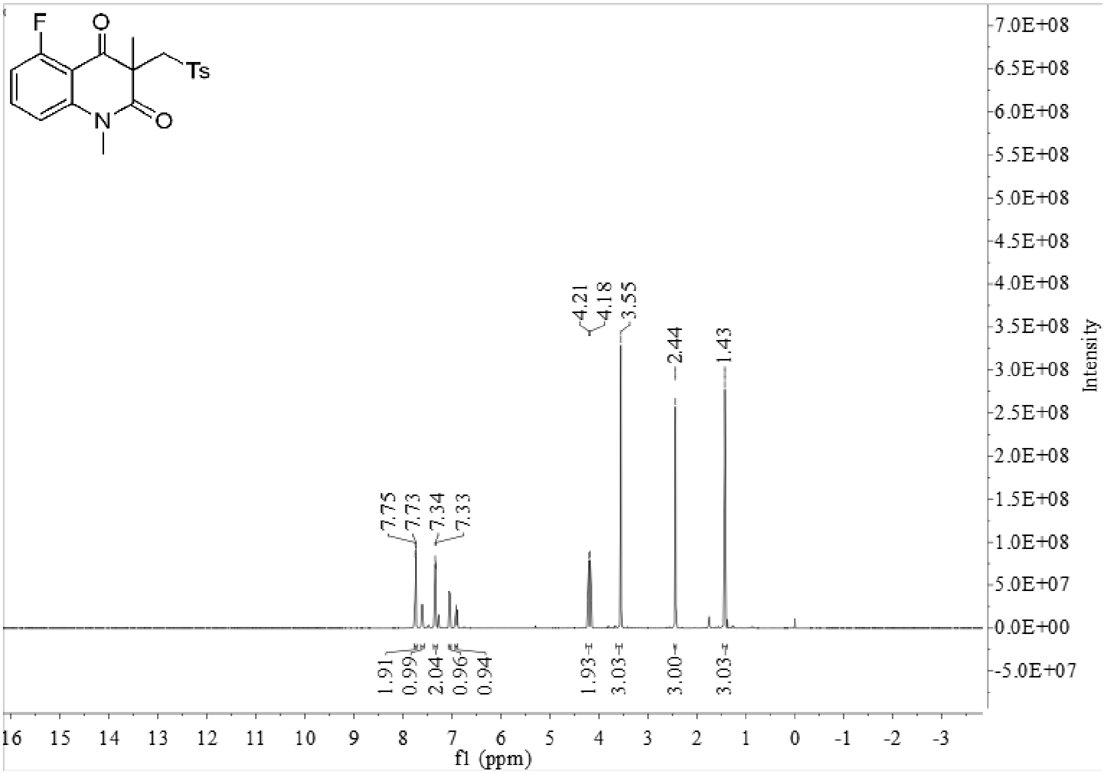
F
O
Ts
N
O
7.75
7.73
7.34
7.33
4.21
4.18
3.55
2.44
1.43
1.91
0.99
2.04
0.96
0.94
1.93
3.03
3.00
3.03
7.0E+08
6.5E+08
6.0E+08
5.5E+08
5.0E+08
4.5E+08
4.0E+08
3.5E+08
3.0E+08
2.5E+08
2.0E+08
1.5E+08
1.0E+08
5.0E+07
0.0E+00
-5.0E+07
Intensity
16 15 14 13 12 11 10 9 8 7 6 5 4 3 2 1 0 -1 -2 -3
f1 (ppm)

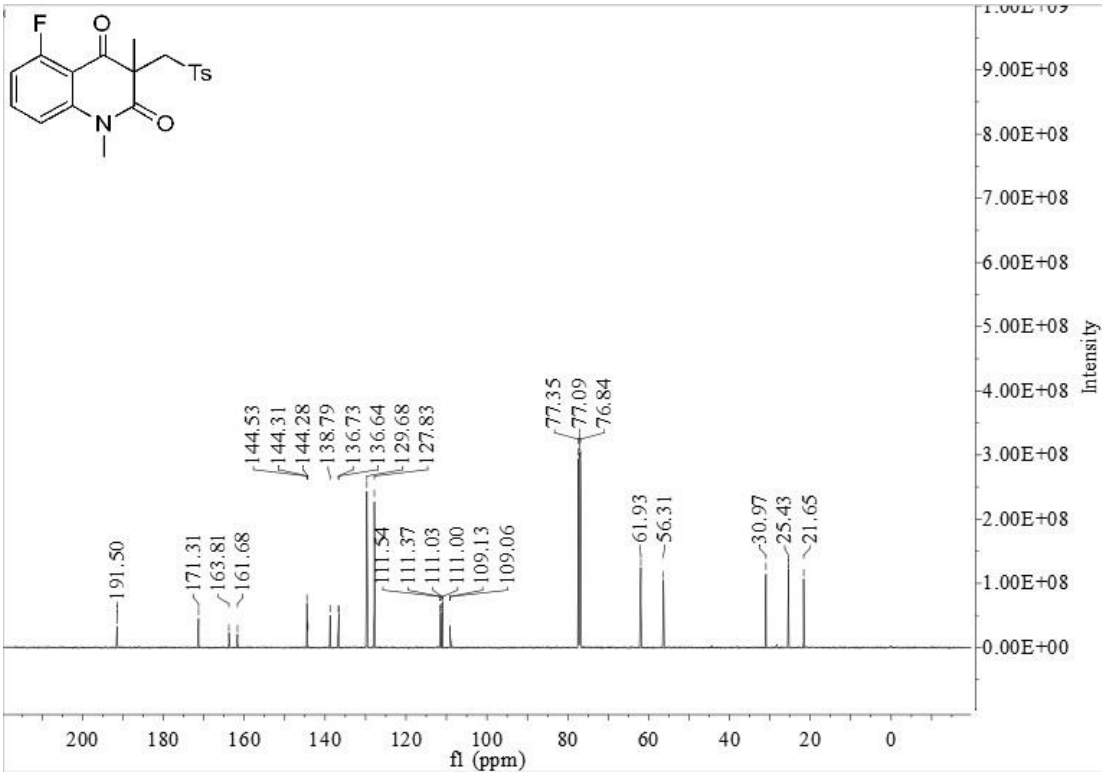
F
O
Ts
N
O
191.50
171.31
163.81
161.68
144.53
144.31
144.28
138.79
136.73
136.64
129.68
127.83
111.54
111.37
111.03
111.00
109.13
109.06
77.35
77.09
76.84
61.93
56.31
30.97
25.43
21.65
9.00E+08
8.00E+08
7.00E+08
6.00E+08
5.00E+08
4.00E+08
3.00E+08
2.00E+08
1.00E+08
0.00E+00
Intensity
200 180 160 140 120 100 80 60 40 20 0
f1 (ppm)

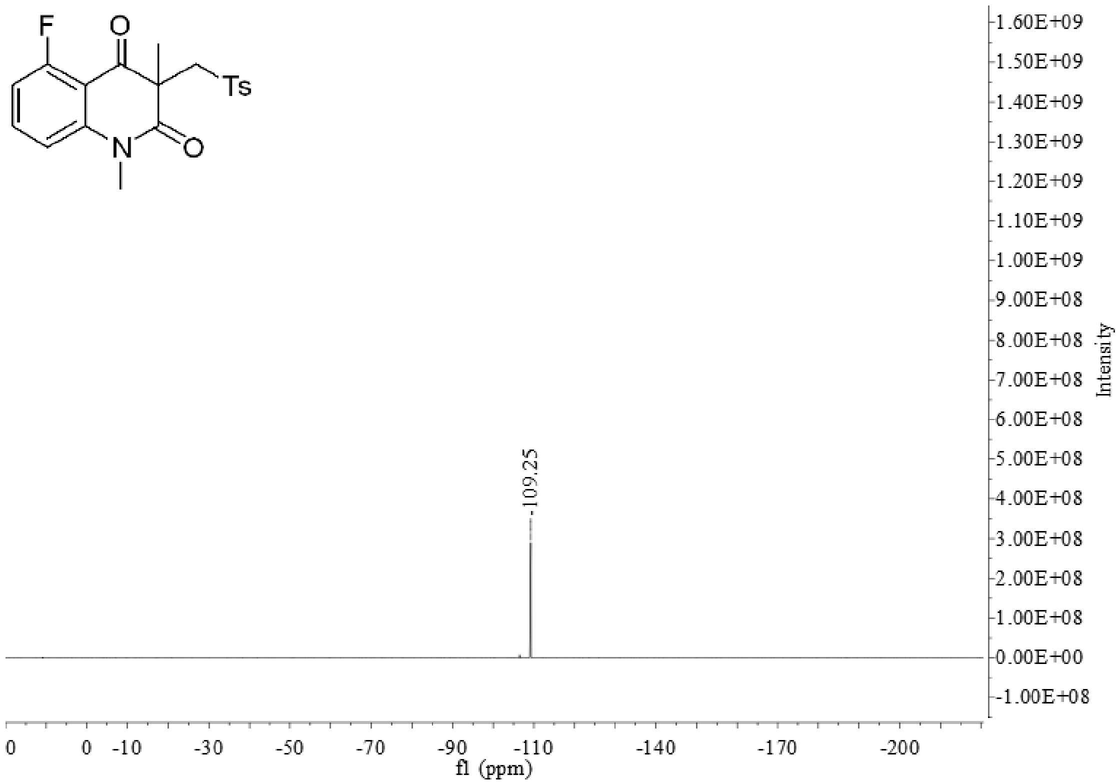

F
O
Ts
N
O
-109.25
1.60E+09
1.50E+09
1.40E+09
1.30E+09
1.20E+09
1.10E+09
1.00E+09
9.00E+08
8.00E+08
7.00E+08
6.00E+08
5.00E+08
4.00E+08
3.00E+08
2.00E+08
1.00E+08
0.00E+00
-1.00E+08
Intensity
0 0 -10 -30 -50 -70 -90 -110 -140 -170 -200
f1 (ppm)

Compound 3c

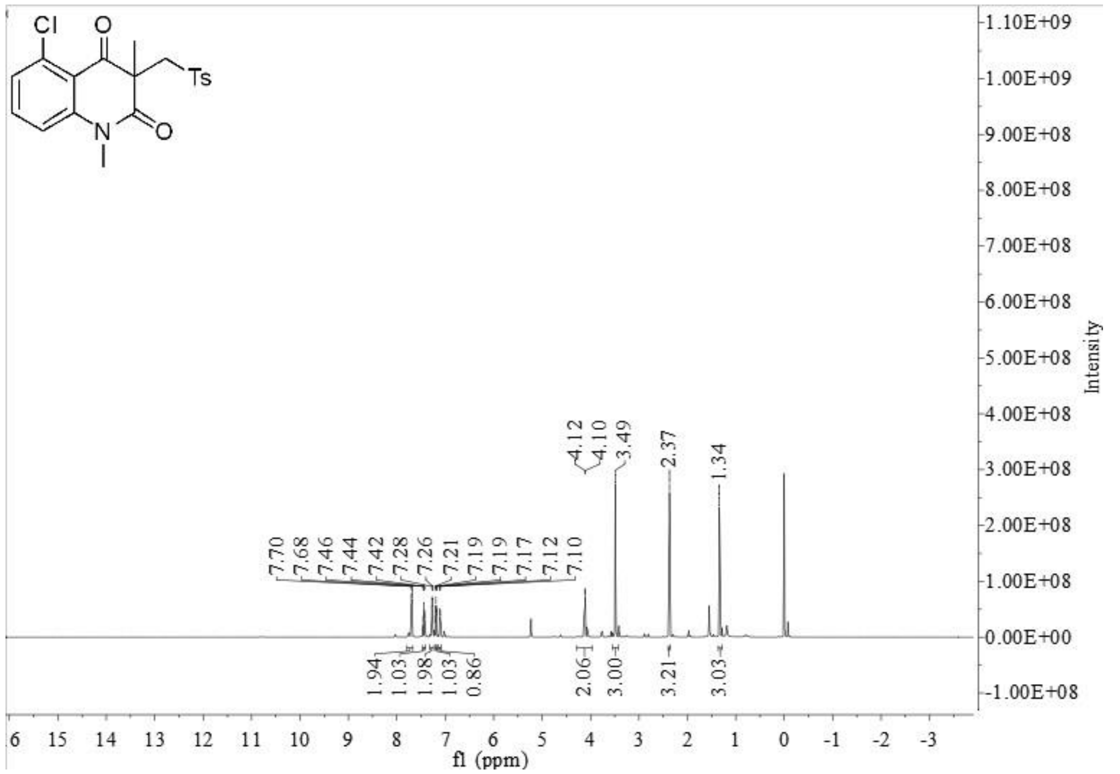

Cl
O
Ts
N
O
7.70 7.68 7.46 7.44 7.42 7.28 7.26 7.21 7.19 7.19 7.17 7.12 7.10
4.12 4.10 3.49
2.37
1.34
1.94 1.03 1.98 1.03 0.86
2.06
3.00
3.21
3.03
1.10E+09
1.00E+09
9.00E+08
8.00E+08
7.00E+08
6.00E+08
5.00E+08
4.00E+08
3.00E+08
2.00E+08
1.00E+08
0.00E+00
-1.00E+08
Intensity
6 15 14 13 12 11 10 9 8 7 6 5 4 3 2 1 0 -1 -2 -3
f1 (ppm)

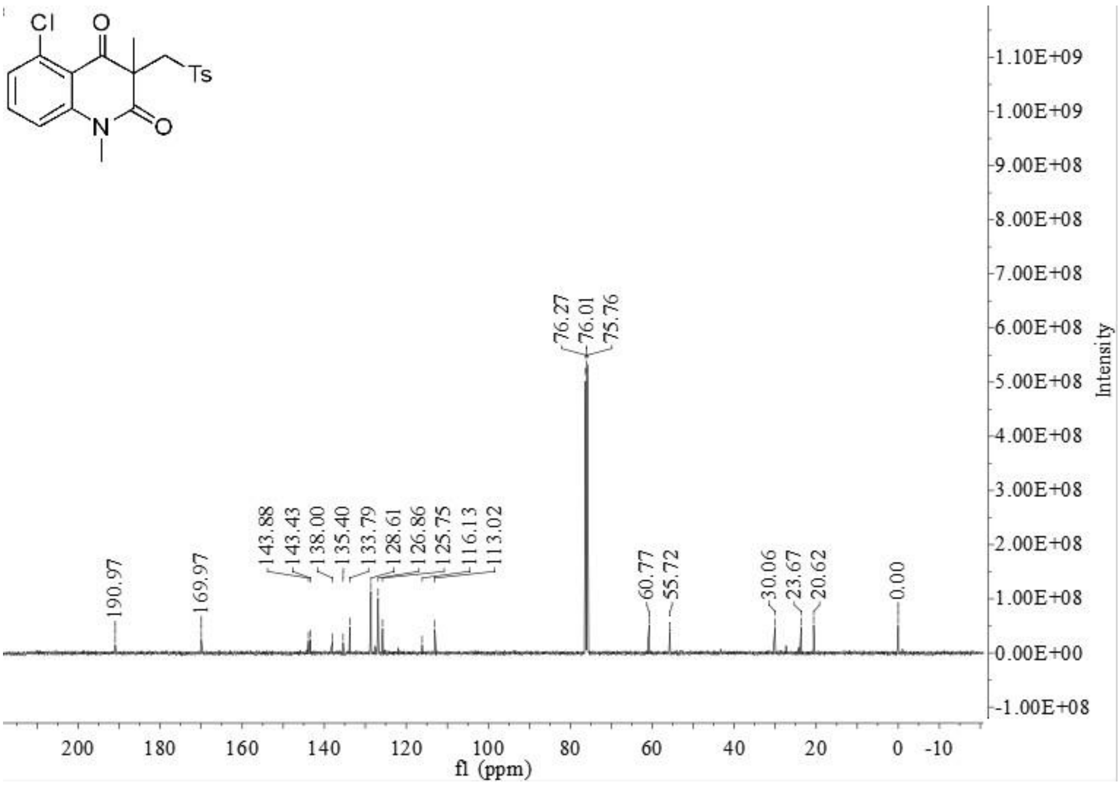
Cl
O
Ts
N
O
190.97
169.97
143.88
143.43
138.00
135.40
133.79
128.61
126.86
125.75
116.13
113.02
76.27
76.01
75.76
60.77
55.72
30.06
23.67
20.62
0.00
1.10E+09
1.00E+09
9.00E+08
8.00E+08
7.00E+08
6.00E+08
5.00E+08
4.00E+08
3.00E+08
2.00E+08
1.00E+08
0.00E+00
-1.00E+08
Intensity
200 180 160 140 120 100 80 60 40 20 0 -10
fl (ppm)

Compound 3d

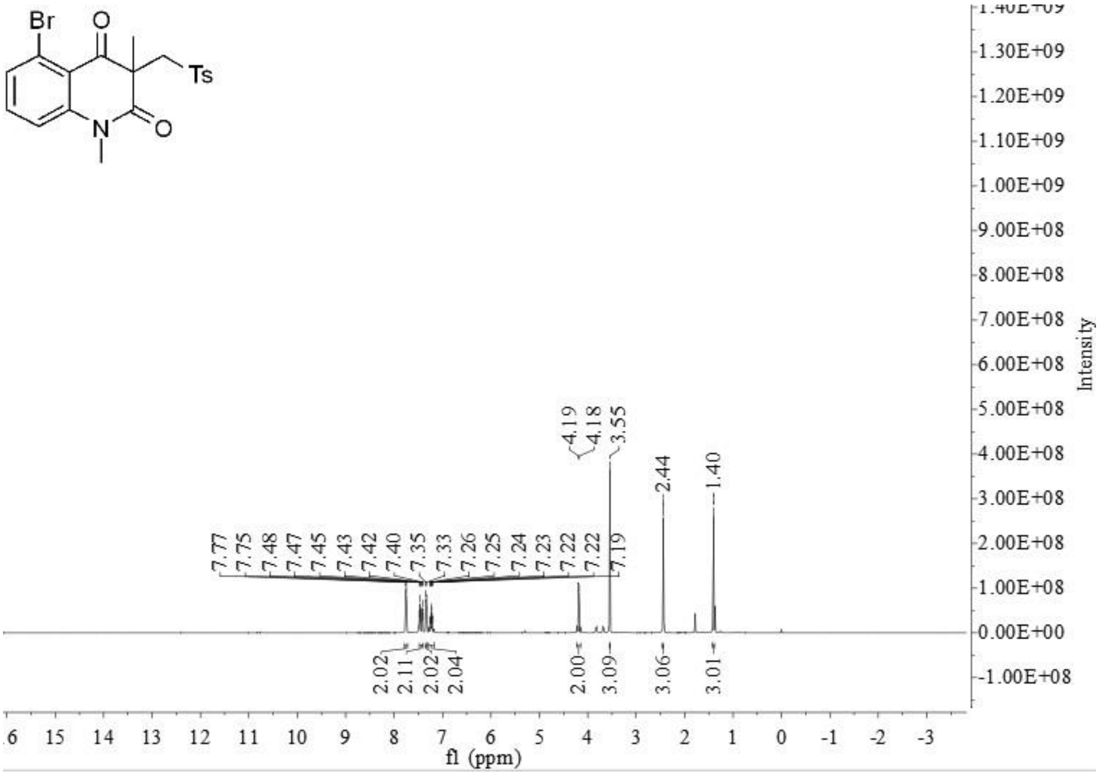
Br
O
Ts
N
O
7.77
7.75
7.48
7.47
7.45
7.43
7.42
7.40
7.35
7.33
7.26
7.25
7.24
7.23
7.22
7.22
7.19
4.19
4.18
3.55
2.44
1.40
2.02
2.11
2.02
2.04
2.00
3.09
3.06
3.01
1.30E+09
1.20E+09
1.10E+09
1.00E+09
9.00E+08
8.00E+08
7.00E+08
6.00E+08
5.00E+08
4.00E+08
3.00E+08
2.00E+08
1.00E+08
0.00E+00
-1.00E+08
Intensity
6 15 14 13 12 11 10 9 8 7 6 5 4 3 2 1 0 -1 -2 -3
fl (ppm)

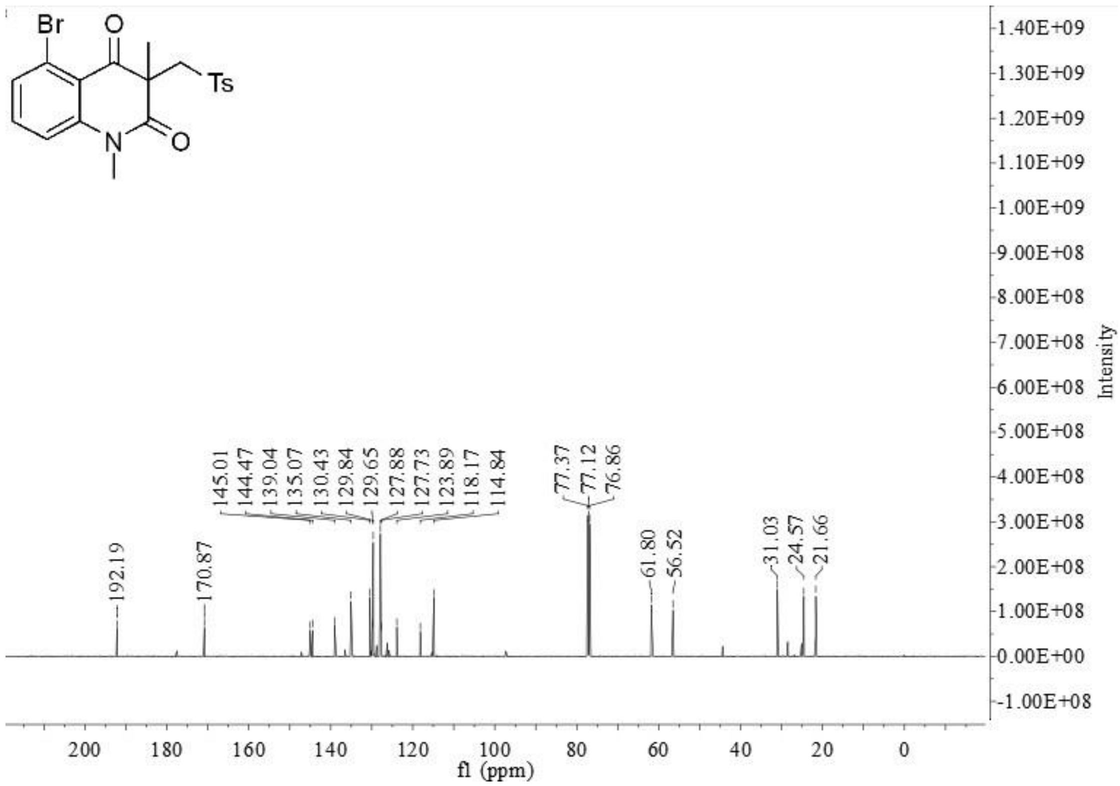
Br
Ts
192.19
170.87
145.01
144.47
139.04
135.07
130.43
129.84
129.65
127.88
127.73
123.89
118.17
114.84
77.37
77.12
76.86
61.80
56.52
31.03
24.57
21.66
fl (ppm)
Intensity

Compound 3e

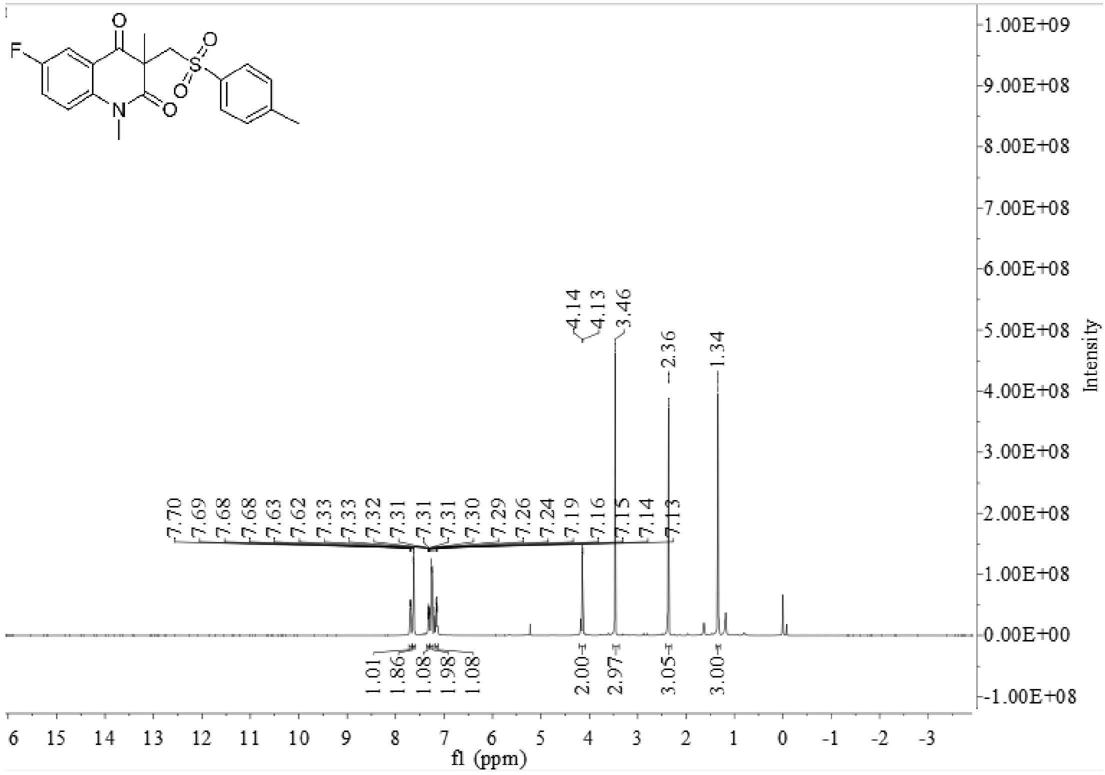
F
7.70
7.69
7.68
7.63
7.62
7.33
7.33
7.32
7.31
7.31
7.31
7.30
7.29
7.26
7.24
7.19
7.16
7.15
7.14
7.13
4.14
4.13
3.46
2.36
1.34
1.01
1.86
1.08
1.98
1.08
2.00
2.97
3.05
3.00
fl (ppm)
Intensity

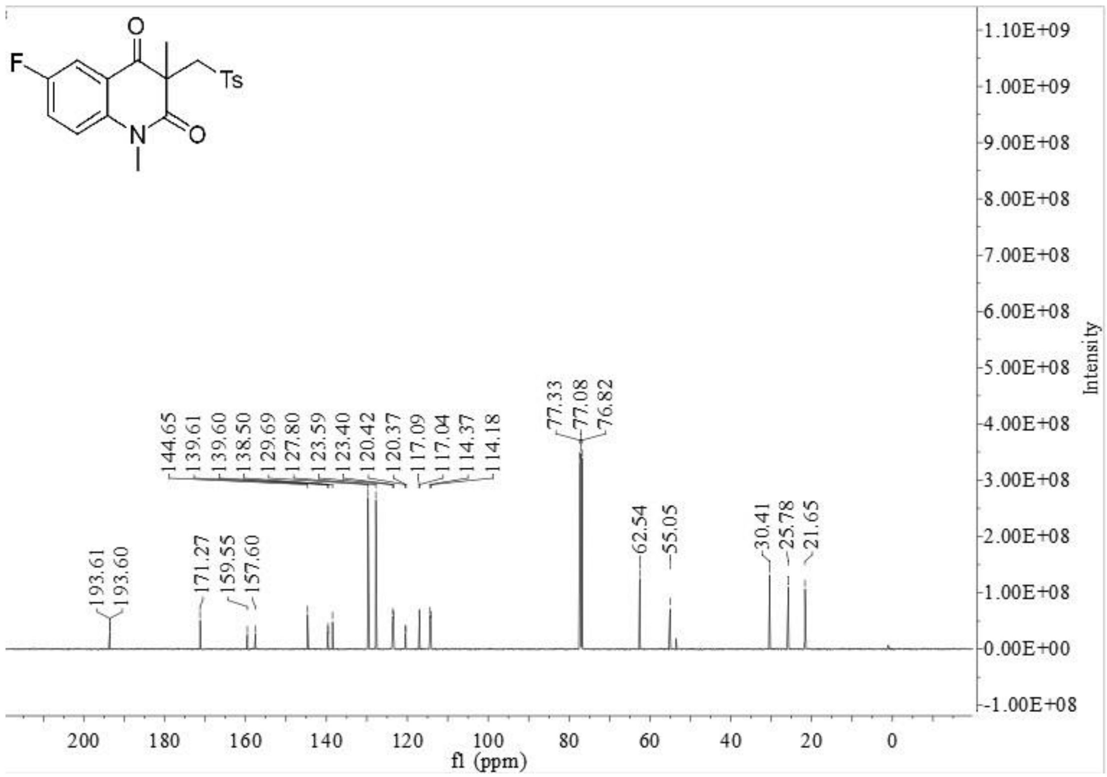
F
O
O
N
Ts
193.61
193.60
171.27
159.55
157.60
144.65
139.61
139.60
138.50
129.69
127.80
123.59
123.40
120.42
120.37
117.09
117.04
114.37
114.18
77.33
77.08
76.82
62.54
55.05
30.41
25.78
21.65
fl (ppm)
Intensity

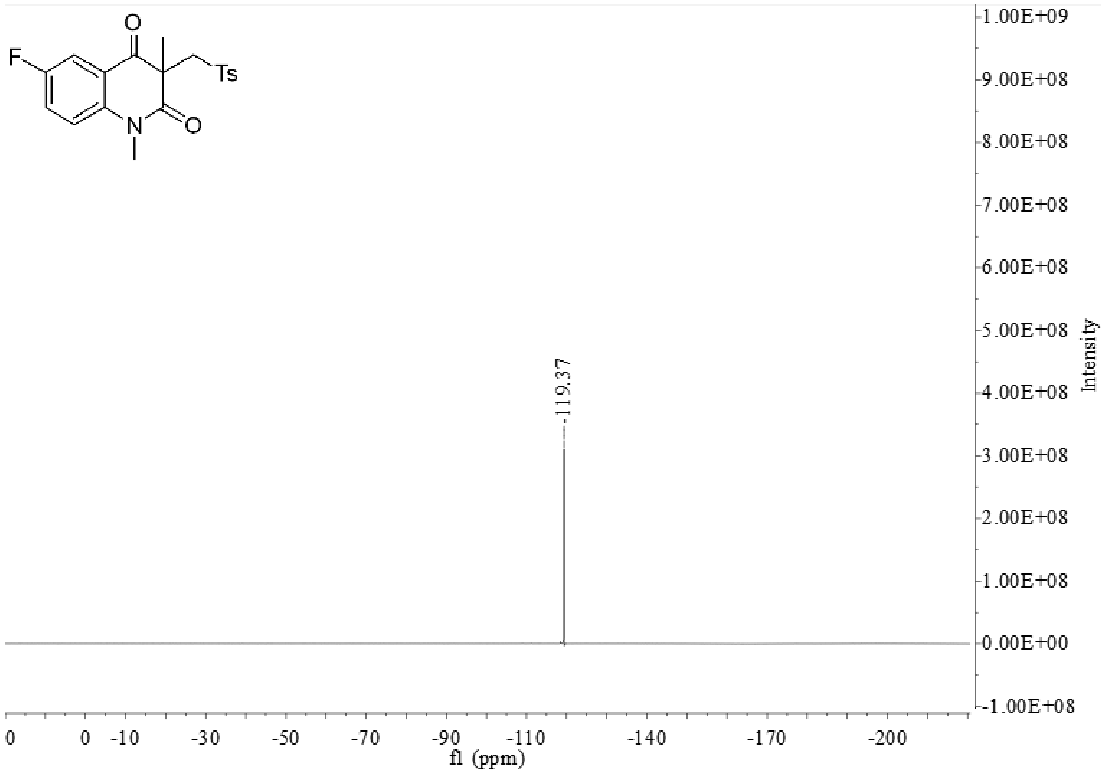
F
O
O
N
Ts
-119.37
fl (ppm)
Intensity

Compound 3f

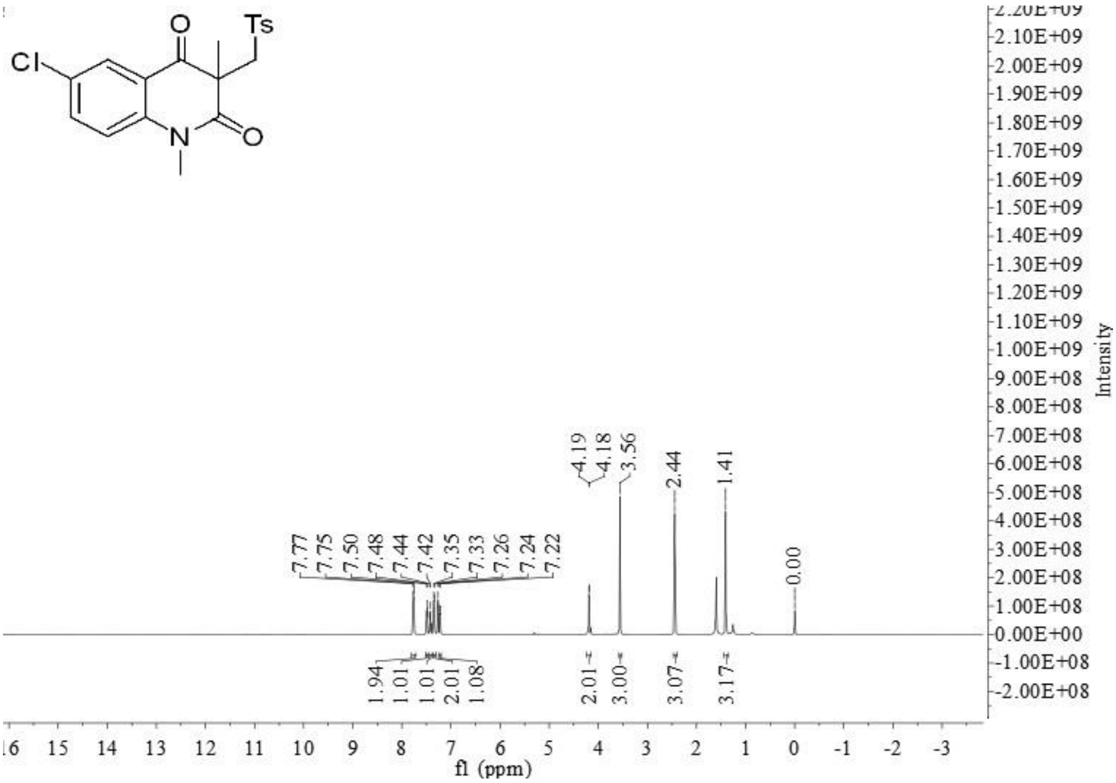
Cl
O
Ts
N
O
7.77
7.75
7.50
7.48
7.44
7.42
7.35
7.33
7.26
7.24
7.22
4.19
4.18
3.56
2.44
1.41
0.00
1.94
1.01
1.01
2.01
1.08
2.01
3.00
3.07
3.17
f1 (ppm)
Intensity

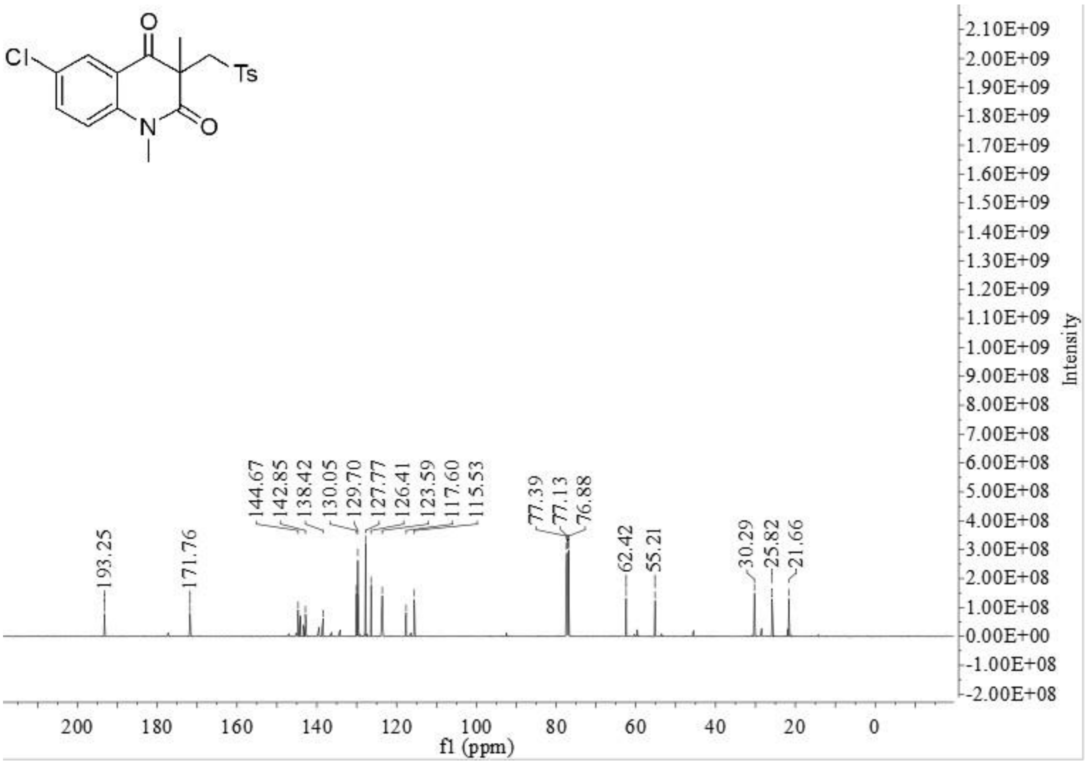
Cl
O
Ts
N
O
193.25
171.76
144.67
142.85
138.42
130.05
129.70
127.77
126.41
123.59
117.60
115.53
77.39
77.13
76.88
62.42
55.21
30.29
25.82
21.66
f1 (ppm)
Intensity

Compound 3g

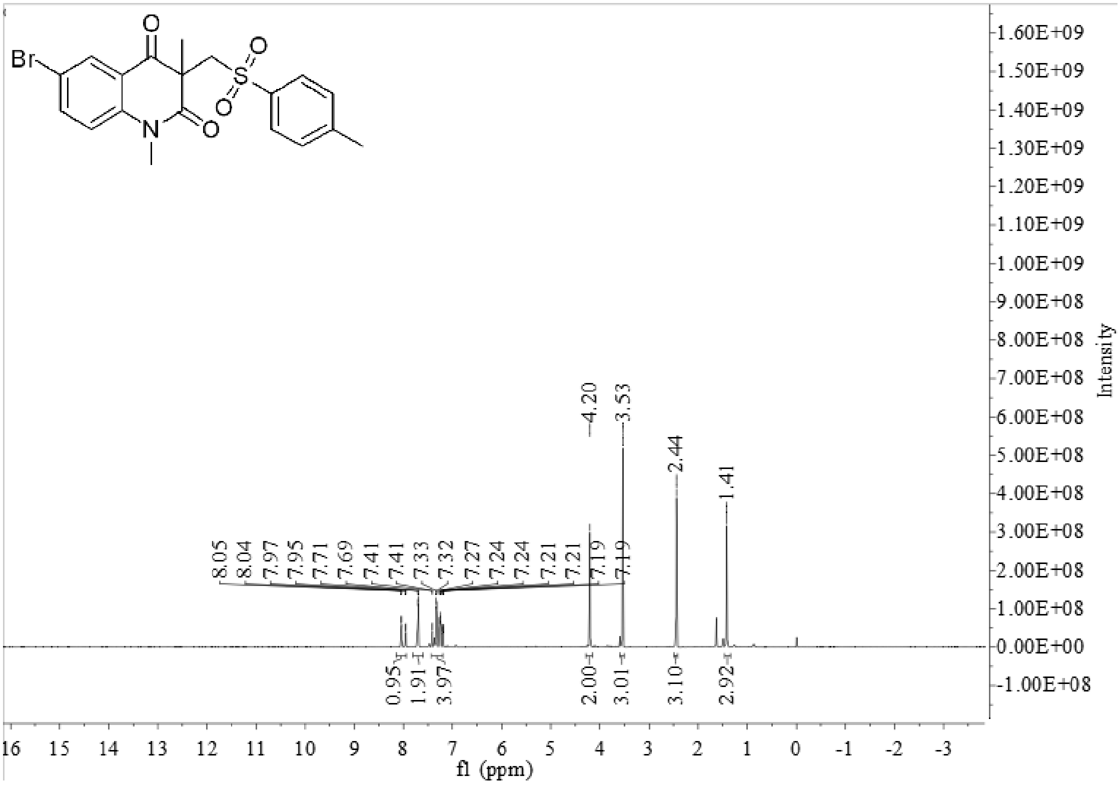

Br
O
N
S
Intensity
fl (ppm)
8.05
8.04
7.97
7.95
7.71
7.69
7.41
7.41
7.33
7.32
7.27
7.24
7.24
7.21
7.21
7.19
7.19
4.20
3.53
2.44
1.41
0.95
1.91
3.97
2.00
3.01
3.10
2.92

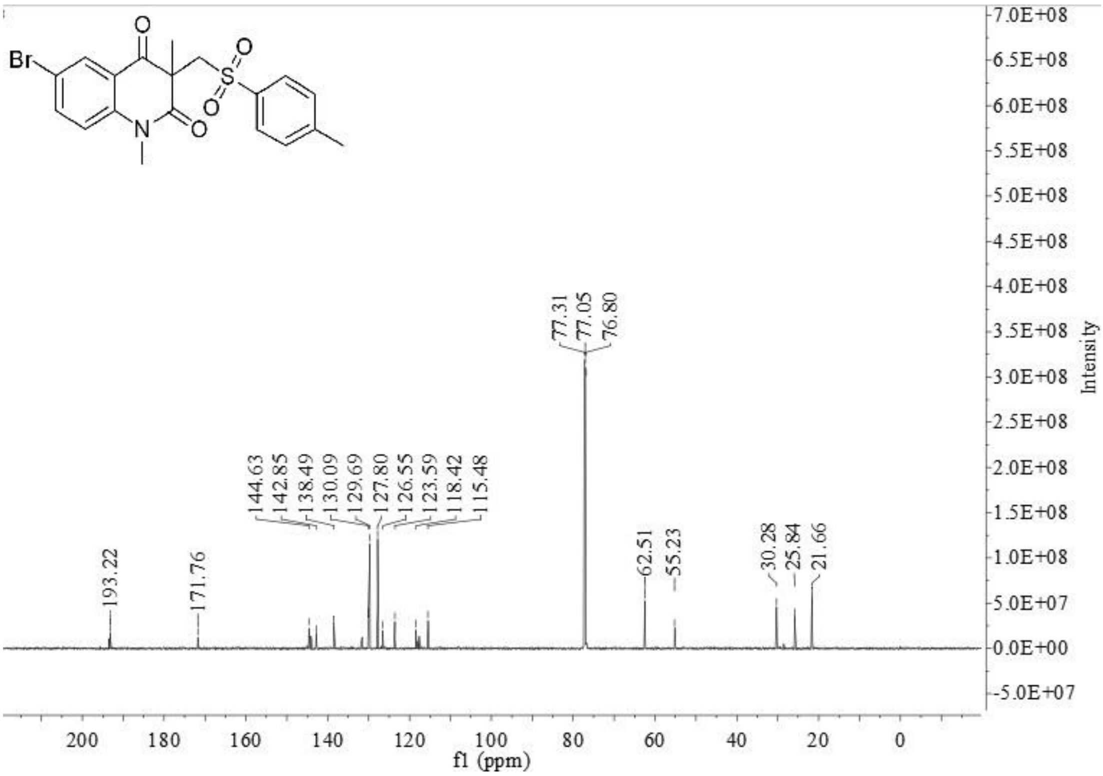

Br
O
N
S
Intensity
fl (ppm)
193.22
171.76
144.63
142.85
138.49
130.09
129.69
127.80
126.55
123.59
118.42
115.48
77.31
77.05
76.80
62.51
55.23
30.28
25.84
21.66

Compound 3h

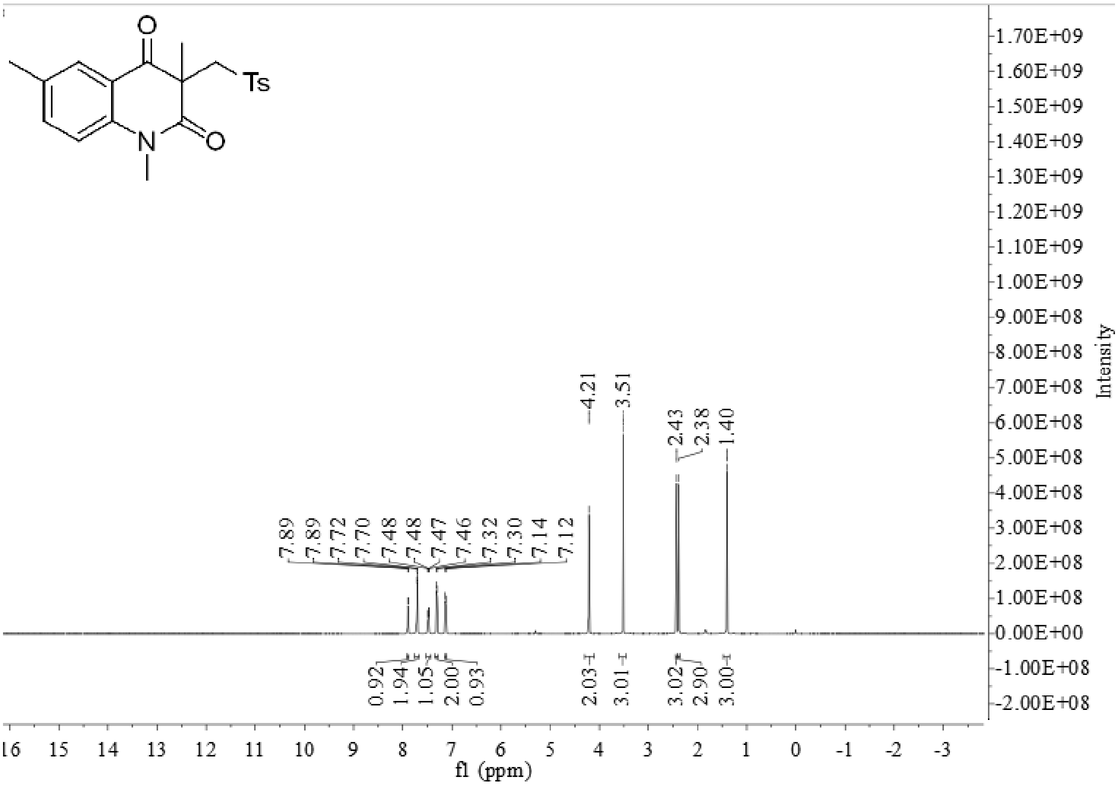
Ts
O
N
7.89
7.89
7.72
7.70
7.48
7.48
7.47
7.46
7.32
7.30
7.14
7.12
4.21
3.51
2.43
2.38
1.40
0.92
1.94
1.05
2.00
0.93
2.03
3.01
3.02
2.90
3.00
fl (ppm)
Intensity

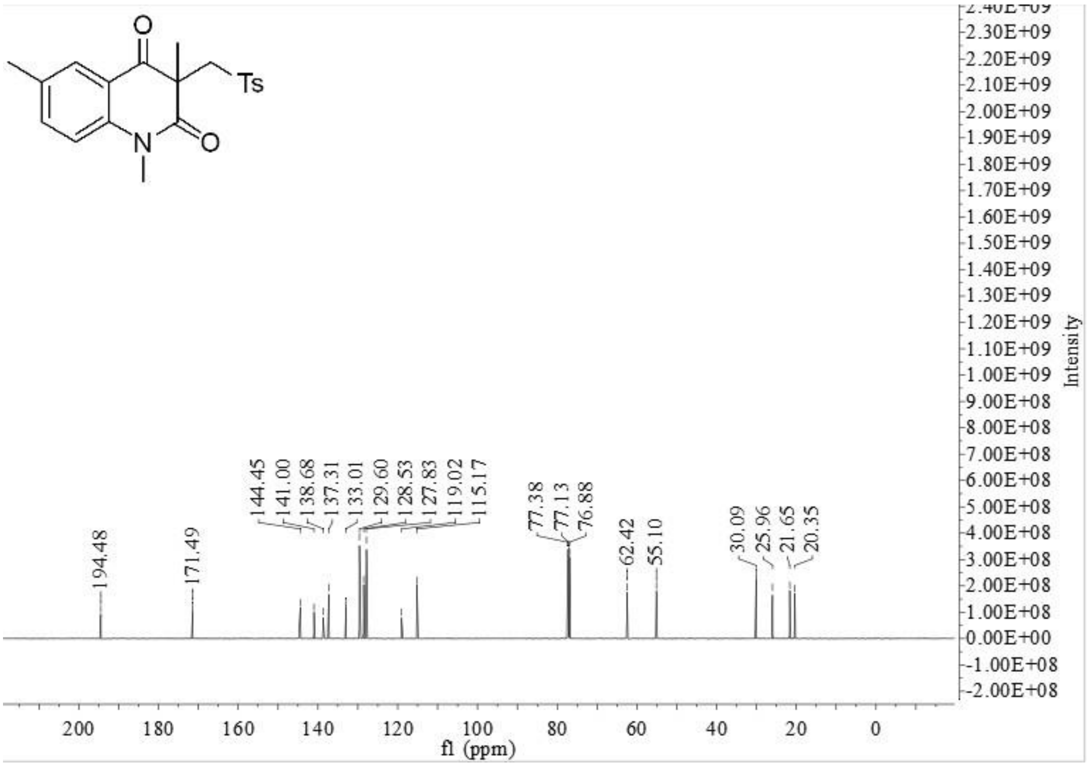
Ts
O
N
194.48
171.49
144.45
141.00
138.68
137.31
133.01
129.60
128.53
127.83
119.02
115.17
77.38
77.13
76.88
62.42
55.10
30.09
25.96
21.65
20.35
fl (ppm)
Intensity

Compound 3i

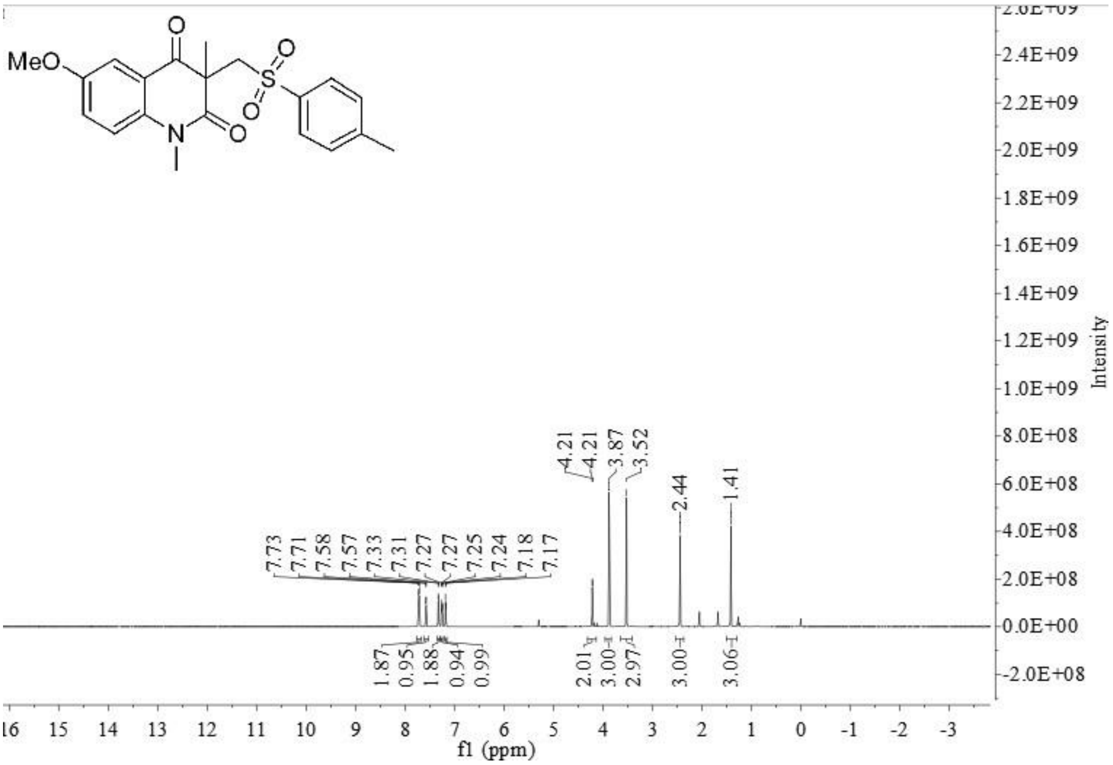

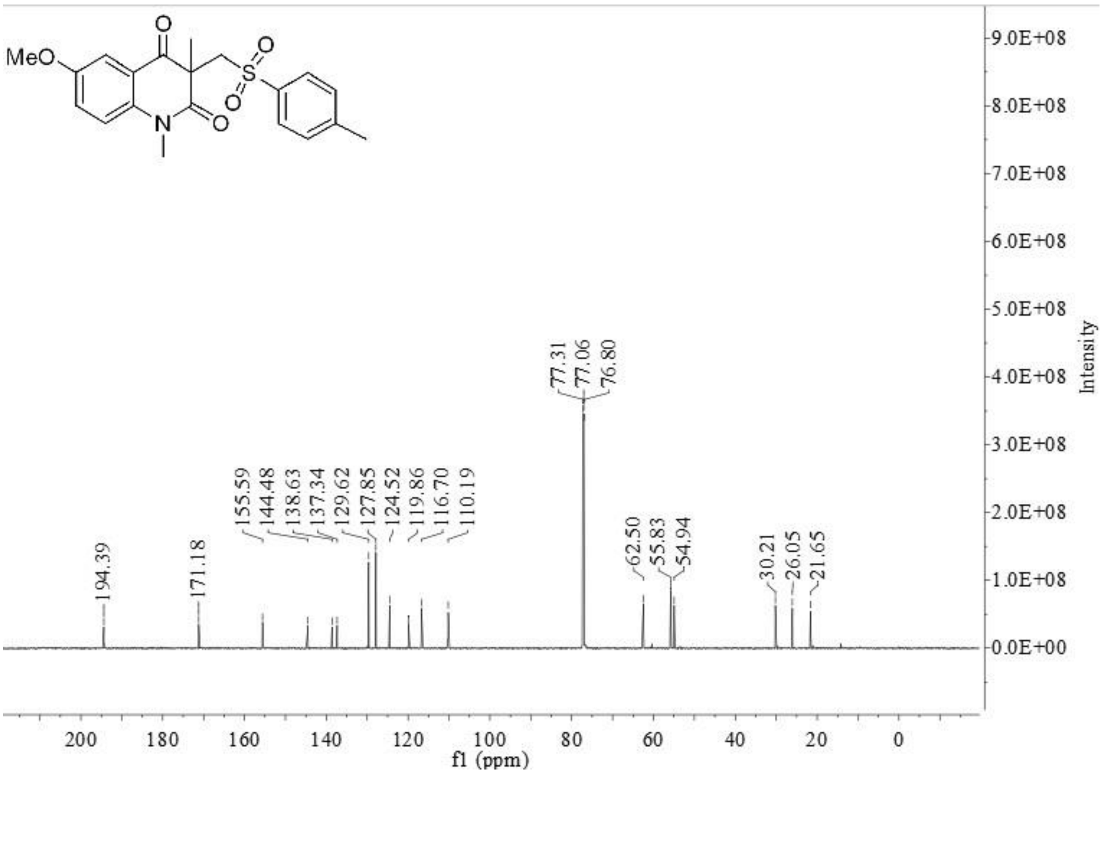

Compound 3j

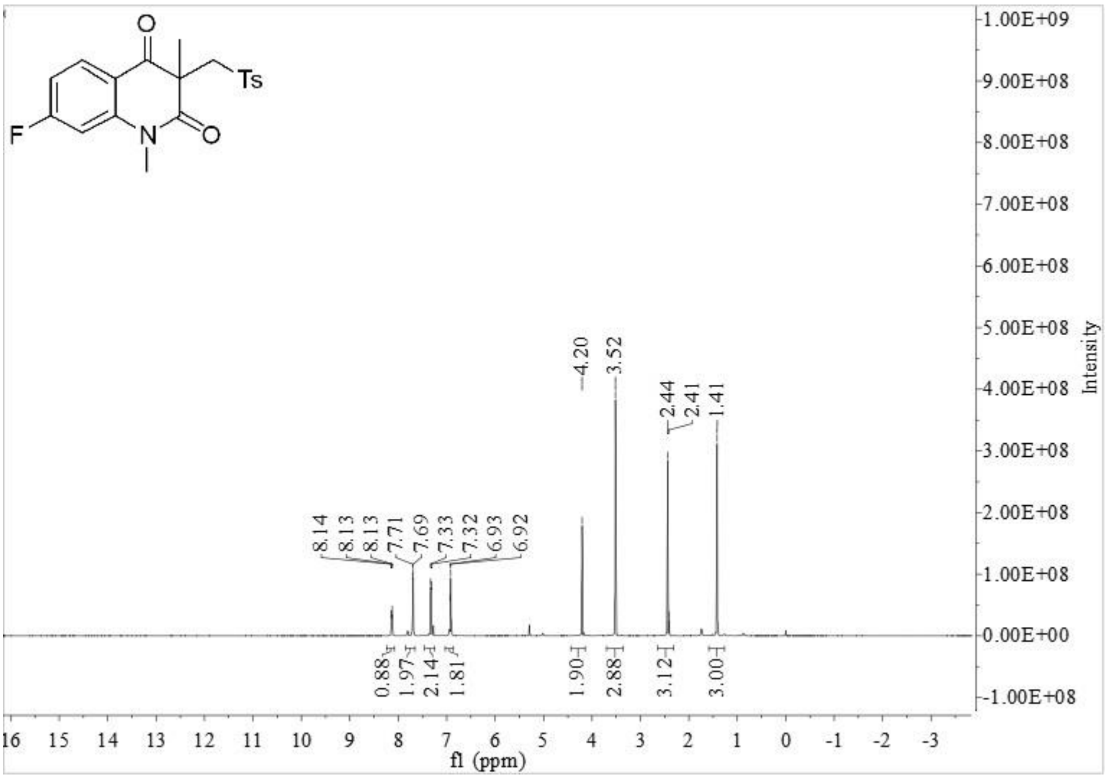

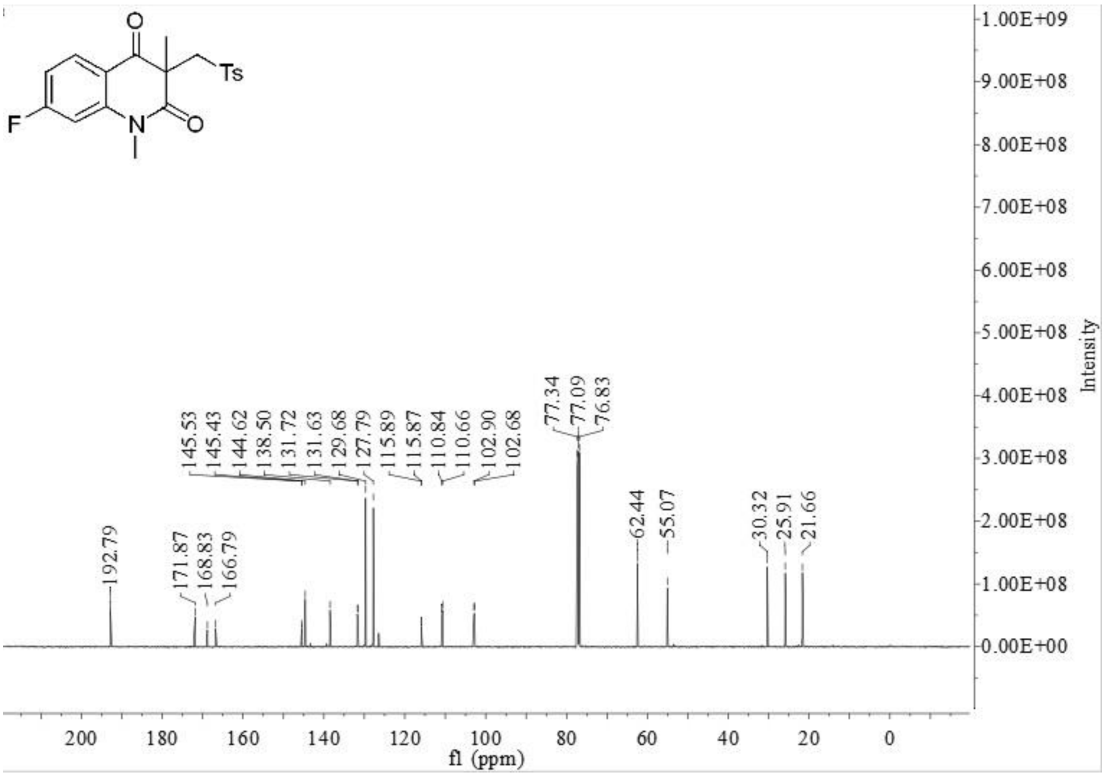

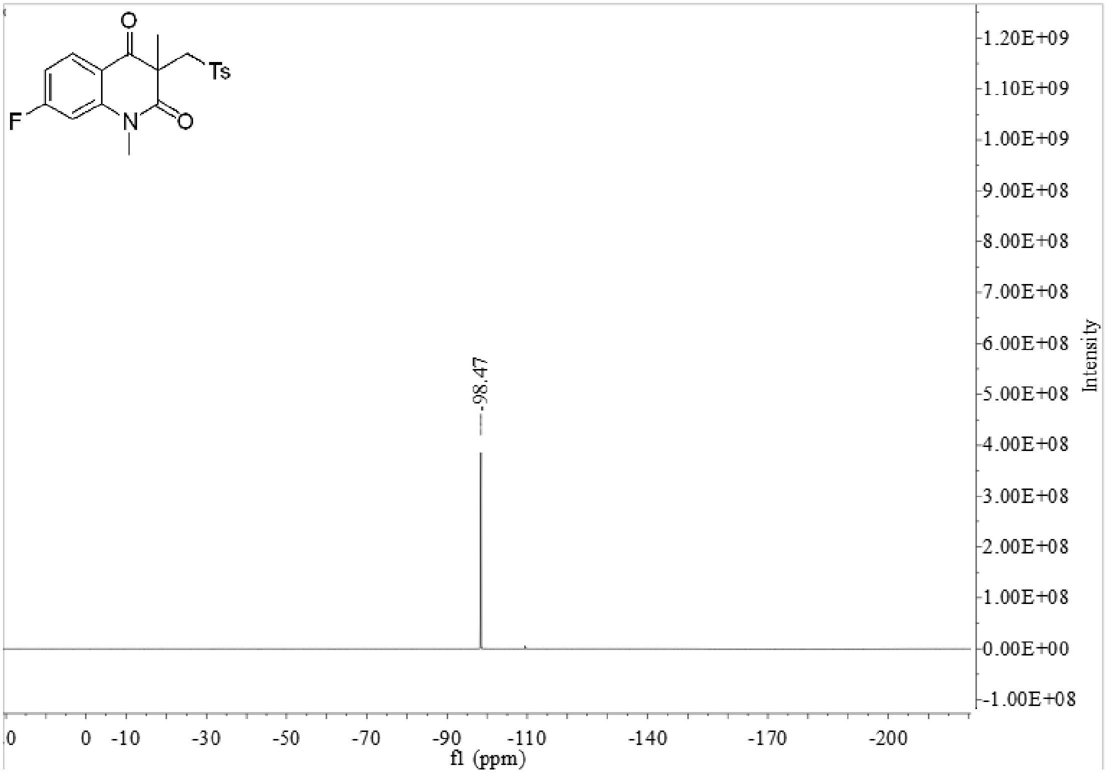
F
N
O
O
Ts
-98.47
1.20E+09
1.10E+09
1.00E+09
9.00E+08
8.00E+08
7.00E+08
6.00E+08
5.00E+08
4.00E+08
3.00E+08
2.00E+08
1.00E+08
0.00E+00
-1.00E+08
Intensity
0 -10 -30 -50 -70 -90 -110 -140 -170 -200
f1 (ppm)

Compound 3k

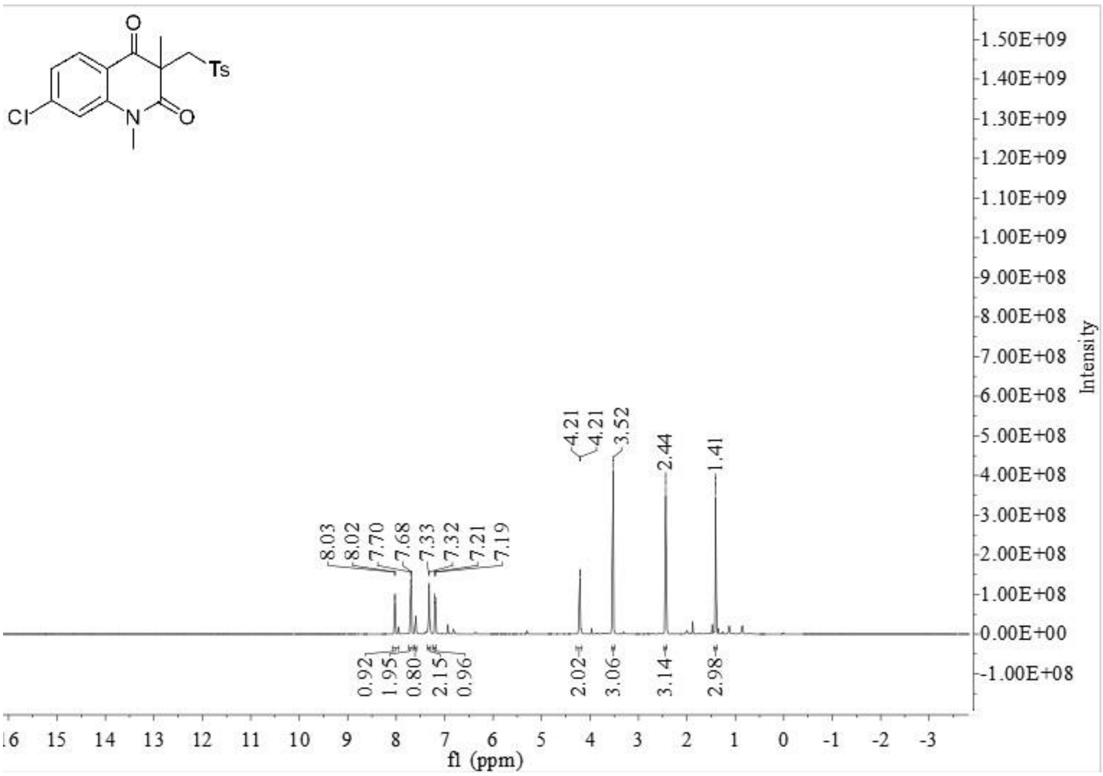
Cl
N
O
O
Ts
8.03
8.02
7.70
7.68
7.33
7.32
7.21
7.19
4.21
4.21
3.52
2.44
1.41
0.92
1.95
0.80
2.15
0.96
2.02
3.06
3.14
2.98
1.50E+09
1.40E+09
1.30E+09
1.20E+09
1.10E+09
1.00E+09
9.00E+08
8.00E+08
7.00E+08
6.00E+08
5.00E+08
4.00E+08
3.00E+08
2.00E+08
1.00E+08
0.00E+00
-1.00E+08
Intensity
16 15 14 13 12 11 10 9 8 7 6 5 4 3 2 1 0 -1 -2 -3
f1 (ppm)

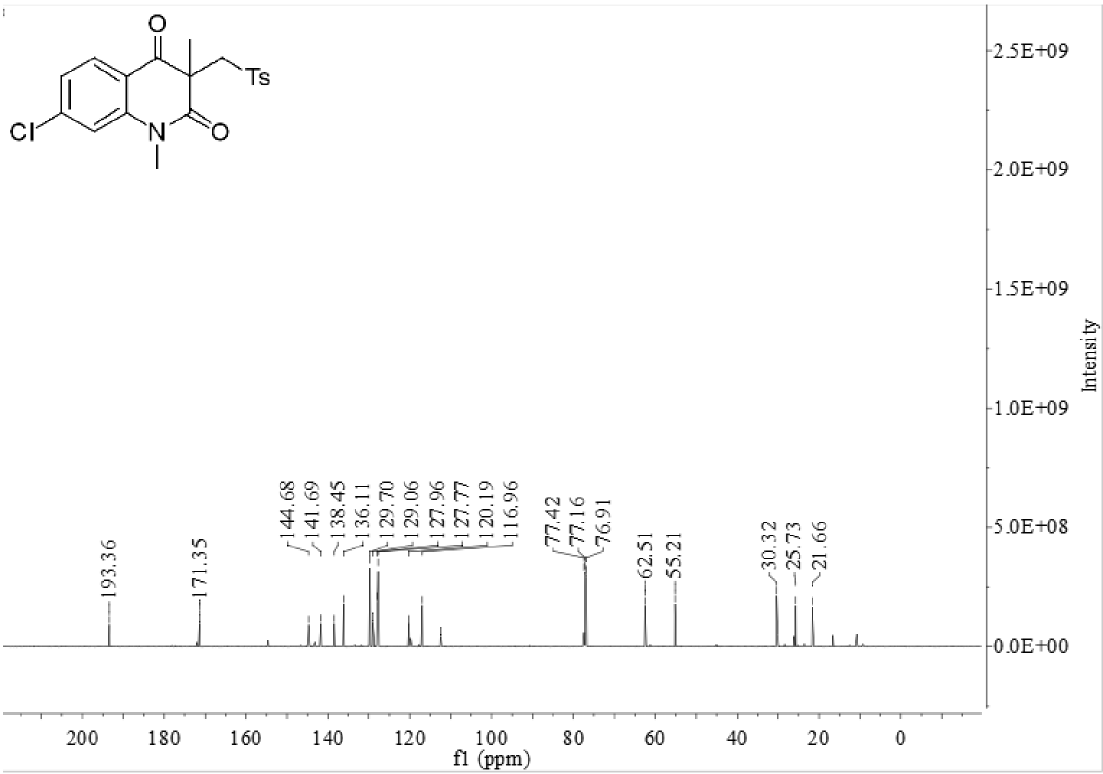
Ts
Cl
N
O
193.36
171.35
144.68
141.69
138.45
136.11
129.70
129.06
127.96
127.77
120.19
116.96
77.42
77.16
76.91
62.51
55.21
30.32
25.73
21.66
2.5E+09
2.0E+09
1.5E+09
1.0E+09
5.0E+08
0.0E+00
Intensity
f1 (ppm)

Compound 3l

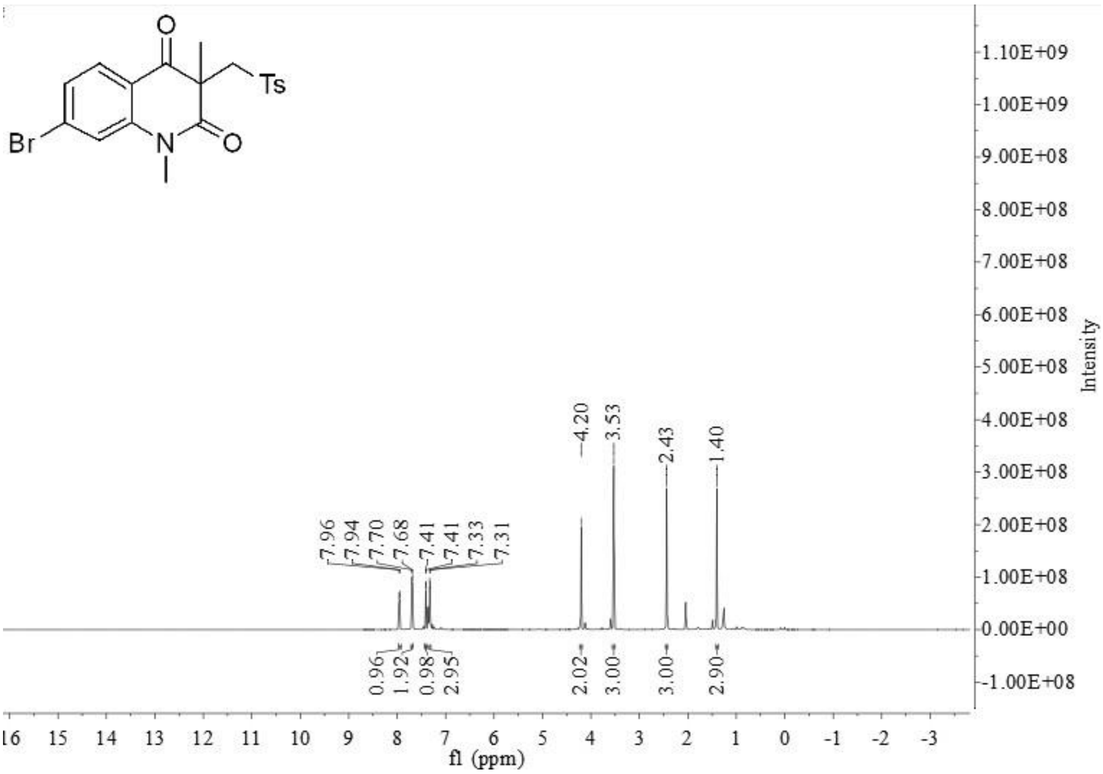
Ts
Br
N
O
7.96
7.94
7.70
7.68
7.41
7.41
7.33
7.31
4.20
3.53
2.43
1.40
0.96
1.92
0.98
2.95
2.02
3.00
3.00
2.90
Intensity
f1 (ppm)

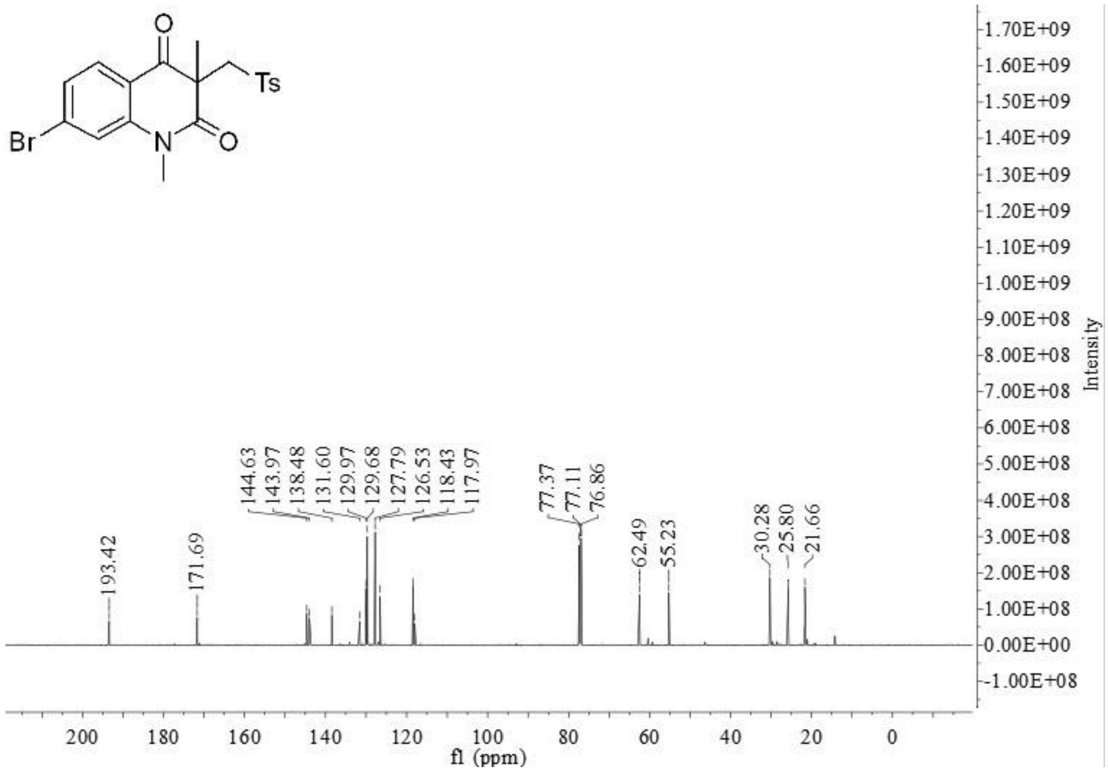
Br
N
O
O
Ts
193.42
171.69
144.63
143.97
138.48
131.60
129.97
129.68
127.79
126.53
118.43
117.97
77.37
77.11
76.86
62.49
55.23
30.28
25.80
21.66
Intensity
f1 (ppm)

Compound 3m

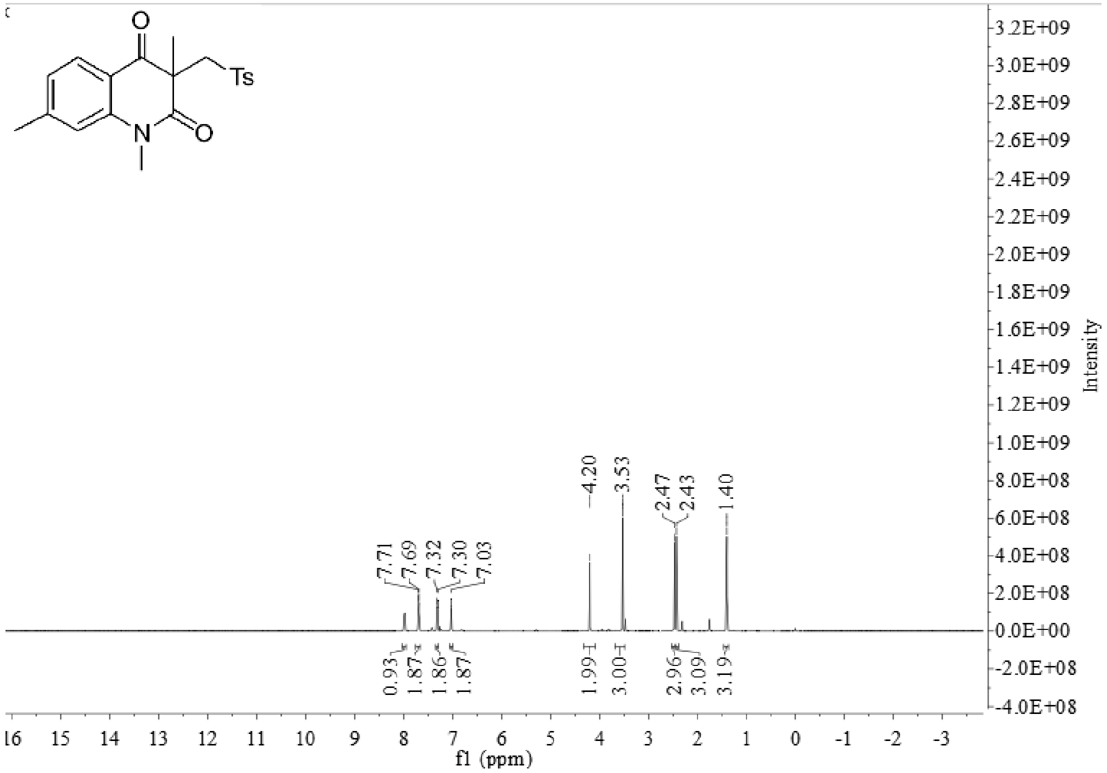
N
O
O
Ts
7.71
7.69
7.32
7.30
7.03
4.20
3.53
2.47
2.43
1.40
0.93
1.87
1.86
1.87
1.99
3.00
2.96
3.09
3.19
Intensity
f1 (ppm)

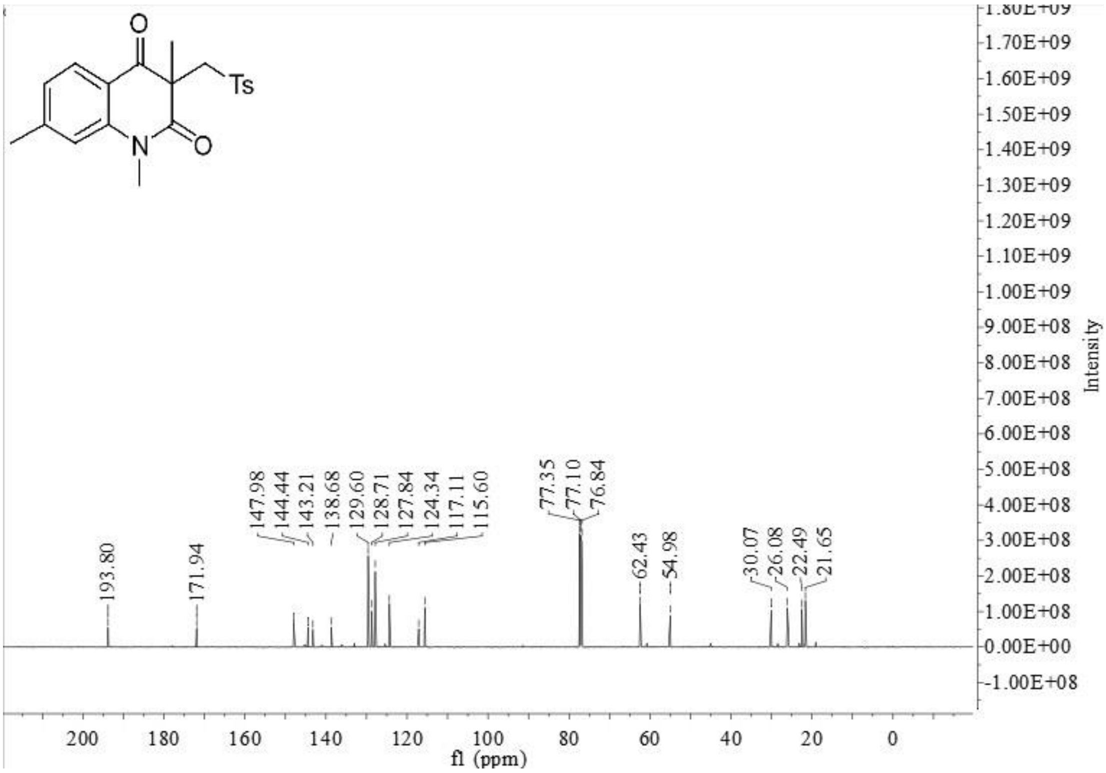
Ts
193.80
171.94
147.98
144.44
143.21
138.68
129.60
128.71
127.84
124.34
117.11
115.60
77.35
77.10
76.84
62.43
54.98
30.07
26.08
22.49
21.65
fl (ppm)
Intensity

Compound 3n

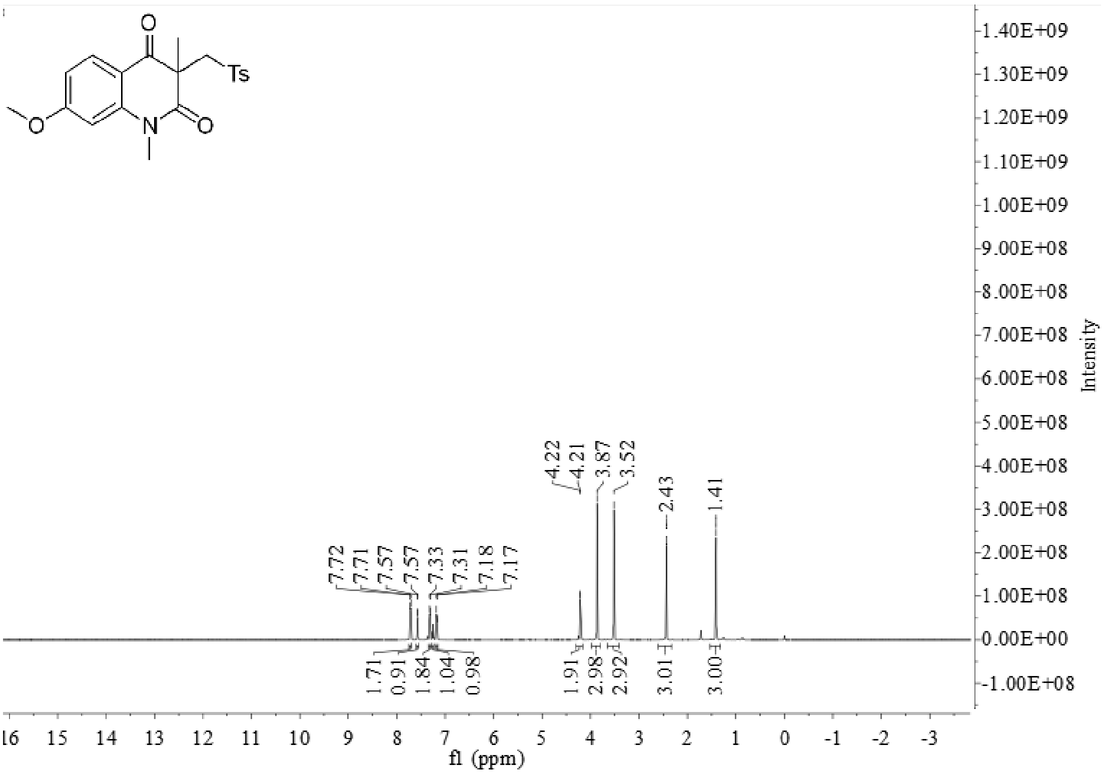
Ts
7.72
7.71
7.57
7.57
7.33
7.31
7.18
7.17
4.22
4.21
3.87
3.52
2.43
1.41
1.71
0.91
1.84
1.04
0.98
1.91
2.98
2.92
3.01
3.00
fl (ppm)
Intensity

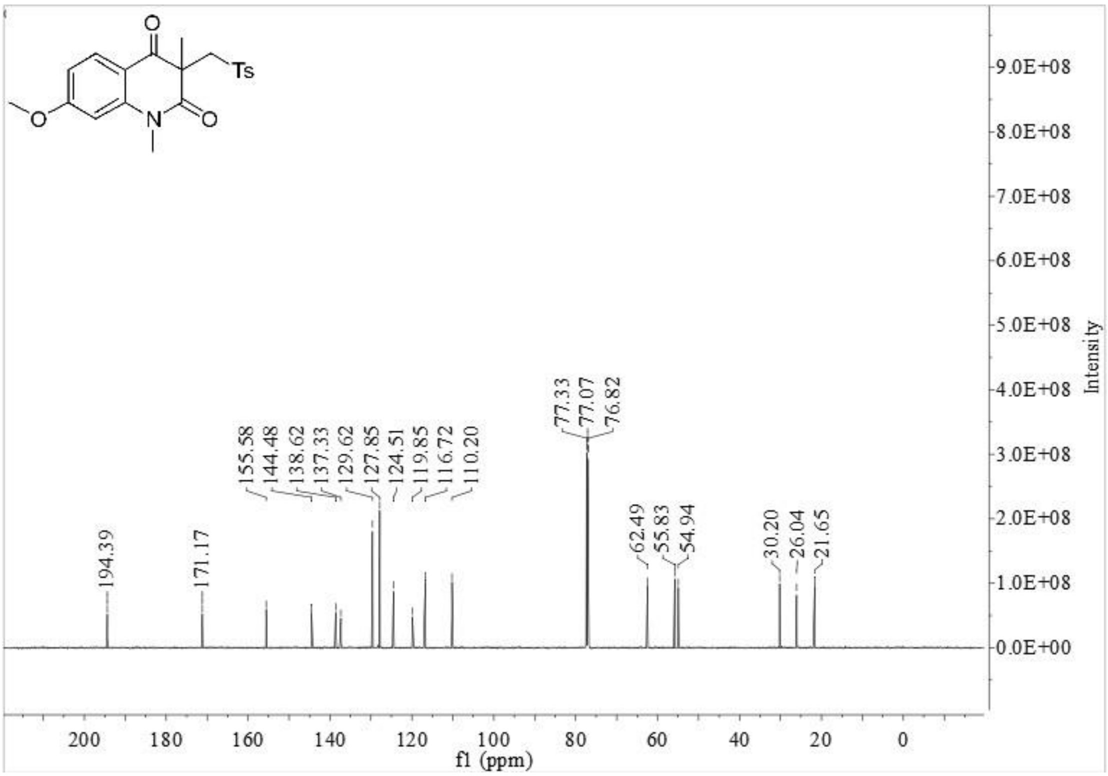
Ts
194.39
171.17
155.58
144.48
138.62
137.33
129.62
127.85
124.51
119.85
116.72
110.20
77.33
77.07
76.82
62.49
55.83
54.94
30.20
26.04
21.65
9.0E+08
8.0E+08
7.0E+08
6.0E+08
5.0E+08
4.0E+08
3.0E+08
2.0E+08
1.0E+08
0.0E+00
Intensity
200 180 160 140 120 100 80 60 40 20 0
f1 (ppm)

Compound 3o

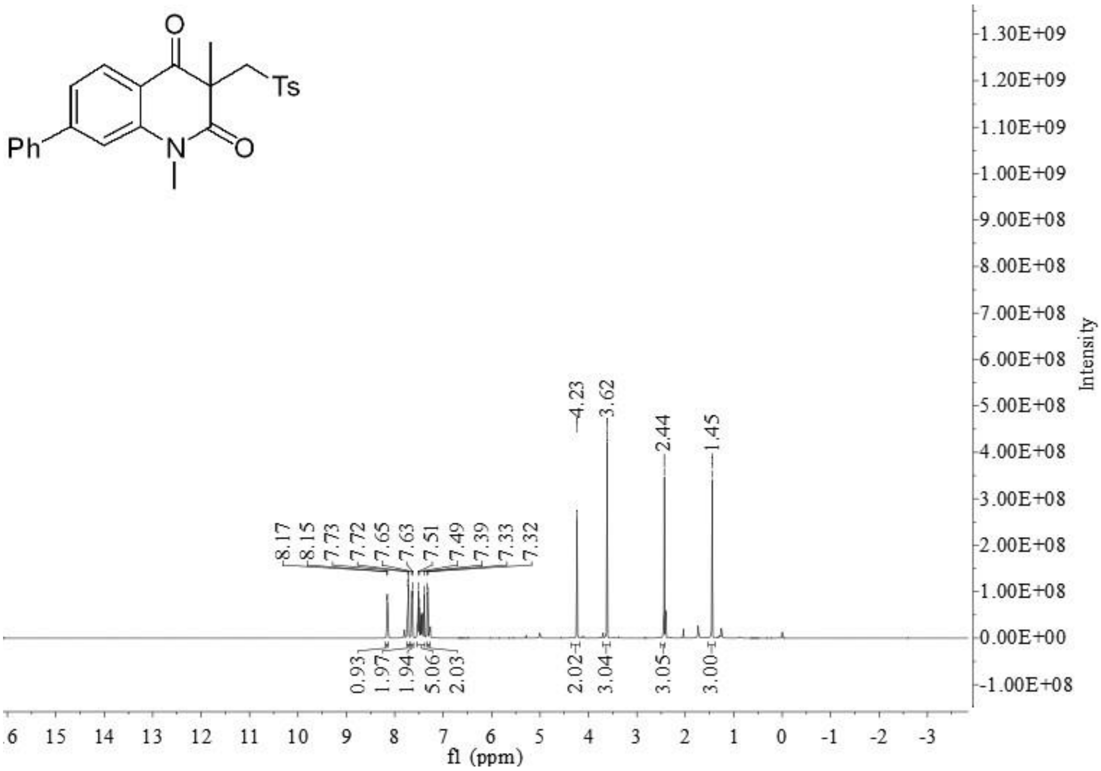
Ts
Ph
8.17
8.15
7.73
7.72
7.65
7.63
7.51
7.49
7.39
7.33
7.32
4.23
3.62
2.44
1.45
0.93
1.97
1.94
5.06
2.03
2.02
3.04
3.05
3.00
1.30E+09
1.20E+09
1.10E+09
1.00E+09
9.00E+08
8.00E+08
7.00E+08
6.00E+08
5.00E+08
4.00E+08
3.00E+08
2.00E+08
1.00E+08
0.00E+00
-1.00E+08
Intensity
6 15 14 13 12 11 10 9 8 7 6 5 4 3 2 1 0 -1 -2 -3
f1 (ppm)

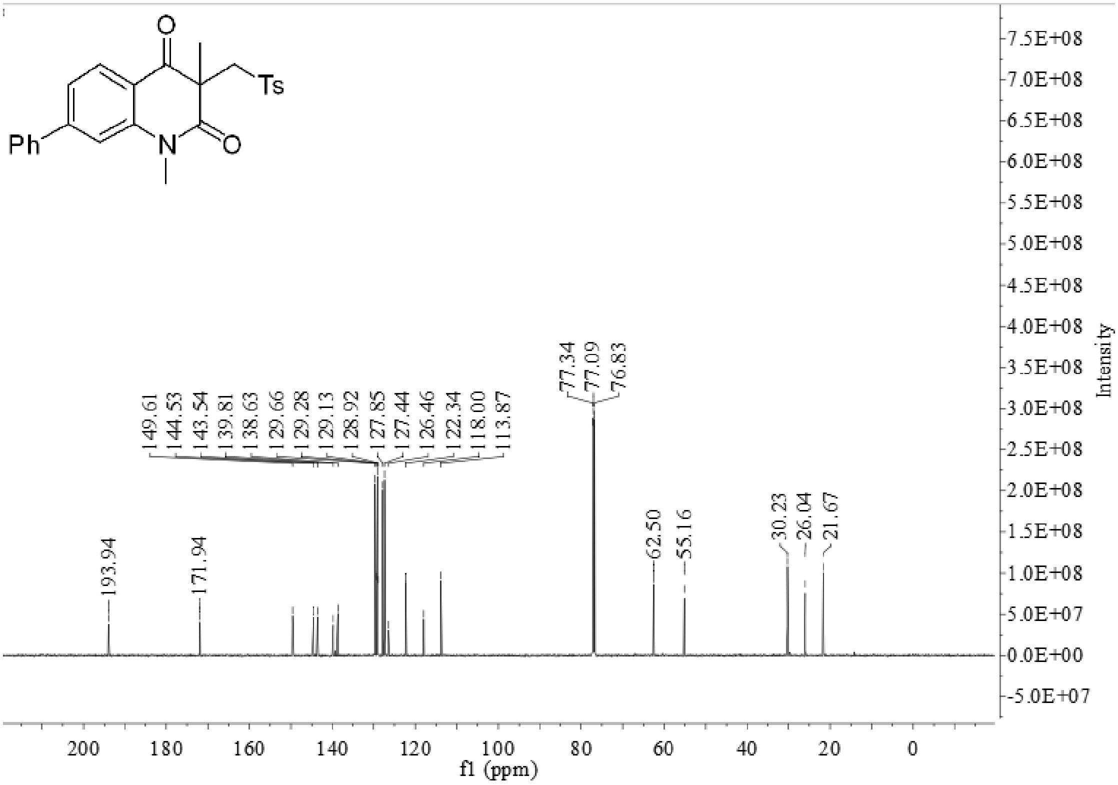
Ph
O
N
O
Ts
193.94
171.94
149.61
144.53
143.54
139.81
138.63
129.66
129.28
129.13
128.92
127.85
127.44
126.46
122.34
118.00
113.87
77.34
77.09
76.83
62.50
55.16
30.23
26.04
21.67
f1 (ppm)
Intensity

Compound 3p

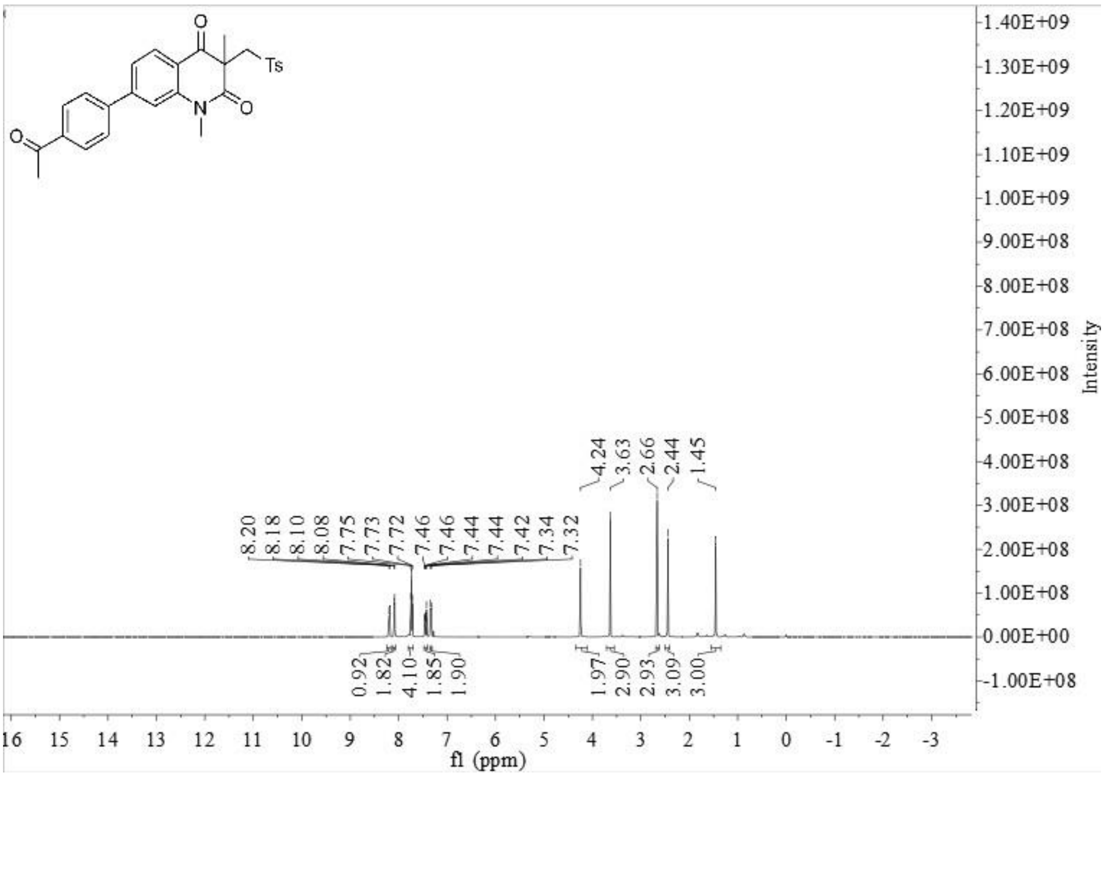
O
N
O
Ts
O
8.20
8.18
8.10
8.08
7.75
7.73
7.72
7.46
7.46
7.44
7.44
7.42
7.34
7.32
4.24
3.63
2.66
2.44
1.45
0.92
1.82
4.10
1.85
1.90
1.97
2.90
2.93
3.09
3.00
f1 (ppm)
Intensity

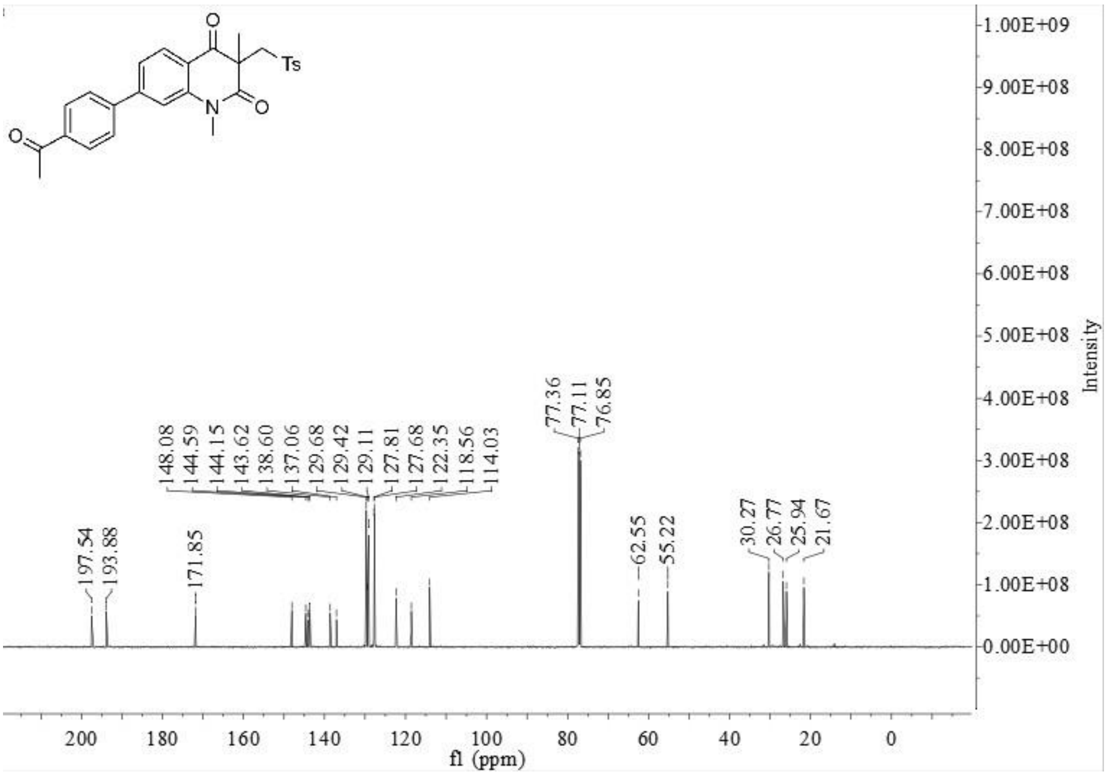
Ts
197.54
193.88
171.85
148.08
144.59
144.15
143.62
138.60
137.06
129.68
129.42
129.11
127.81
127.68
122.35
118.56
114.03
77.36
77.11
76.85
62.55
55.22
30.27
26.77
25.94
21.67
fl (ppm)
Intensity

Compound 3q

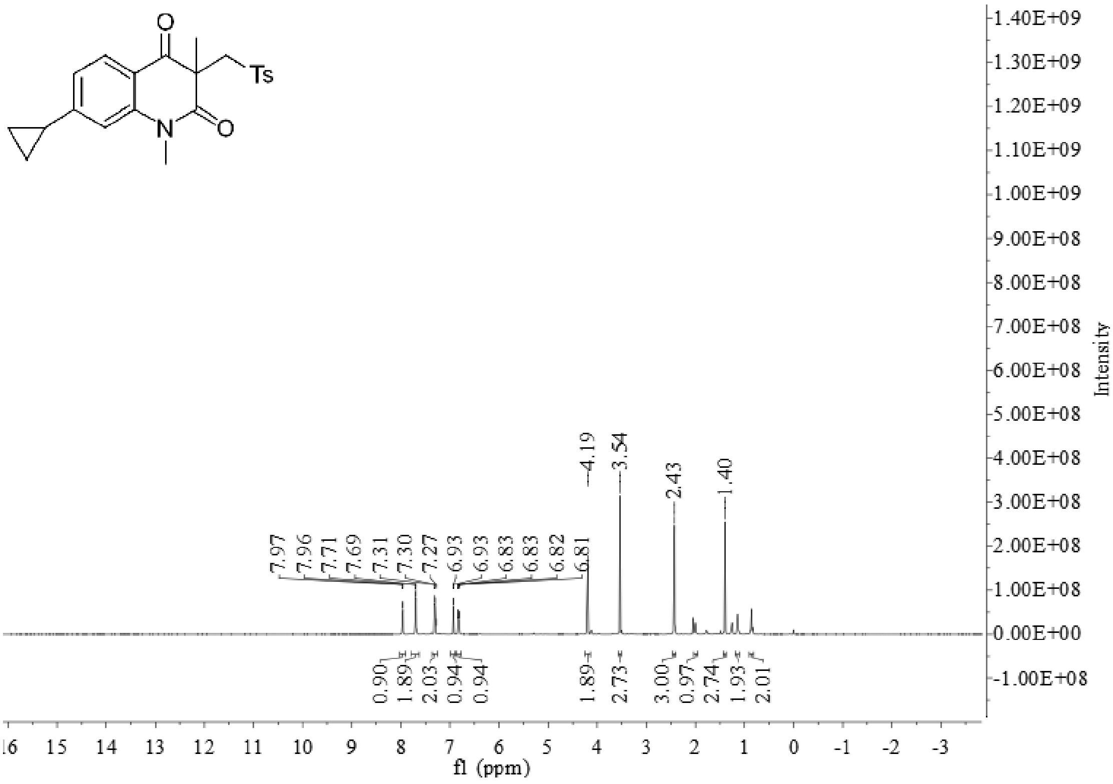
Ts
7.97
7.96
7.71
7.69
7.31
7.30
7.27
6.93
6.93
6.83
6.83
6.82
6.81
4.19
3.54
2.43
1.40
0.90
1.89
2.03
0.94
0.94
1.89
2.73
3.00
0.97
2.74
1.93
2.01
fl (ppm)
Intensity

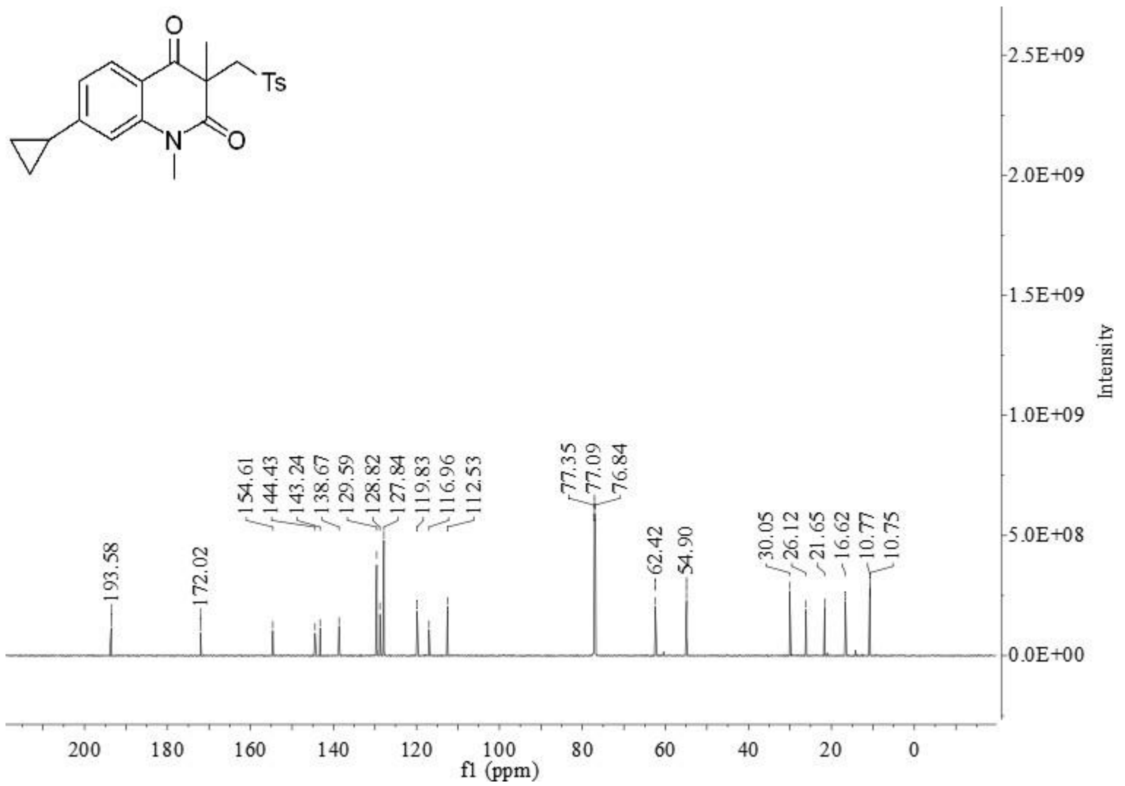
Ts
O
O
N
193.58
172.02
154.61
144.43
143.24
138.67
129.59
128.82
127.84
119.83
116.96
112.53
77.35
77.09
76.84
62.42
54.90
30.05
26.12
21.65
16.62
10.77
10.75
2.5E+09
2.0E+09
1.5E+09
1.0E+09
5.0E+08
0.0E+00
Intensity
200 180 160 140 120 100 80 60 40 20 0
f1 (ppm)

Compound 3r

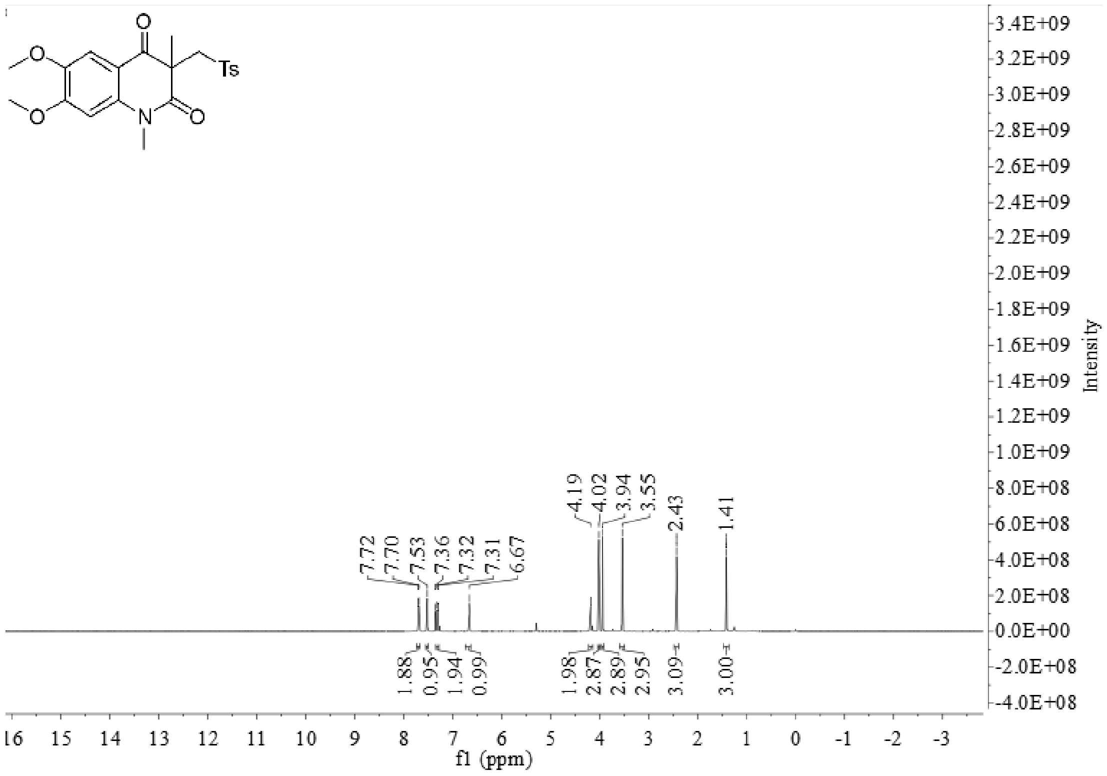
Ts
O
O
N
O
O
7.72
7.70
7.53
7.36
7.32
7.31
6.67
4.19
4.02
3.94
3.55
2.43
1.41
1.88
0.95
1.94
0.99
1.98
2.87
2.89
2.95
3.09
3.00
3.4E+09
3.2E+09
3.0E+09
2.8E+09
2.6E+09
2.4E+09
2.2E+09
2.0E+09
1.8E+09
1.6E+09
1.4E+09
1.2E+09
1.0E+09
8.0E+08
6.0E+08
4.0E+08
2.0E+08
0.0E+00
-2.0E+08
-4.0E+08
Intensity
16 15 14 13 12 11 10 9 8 7 6 5 4 3 2 1 0 -1 -2 -3
f1 (ppm)

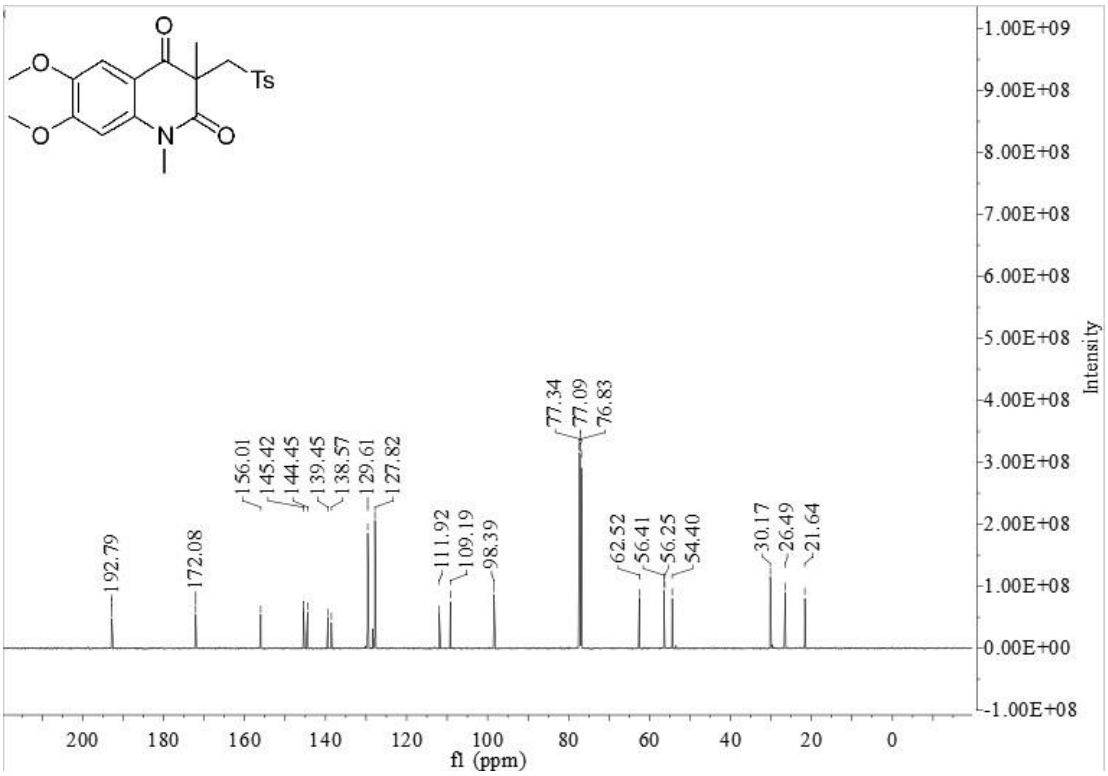
Ts
N
192.79
172.08
156.01
145.42
144.45
139.45
138.57
129.61
127.82
111.92
109.19
98.39
77.34
77.09
76.83
62.52
56.41
56.25
54.40
30.17
26.49
21.64
fl (ppm)
Intensity

Compound 3s

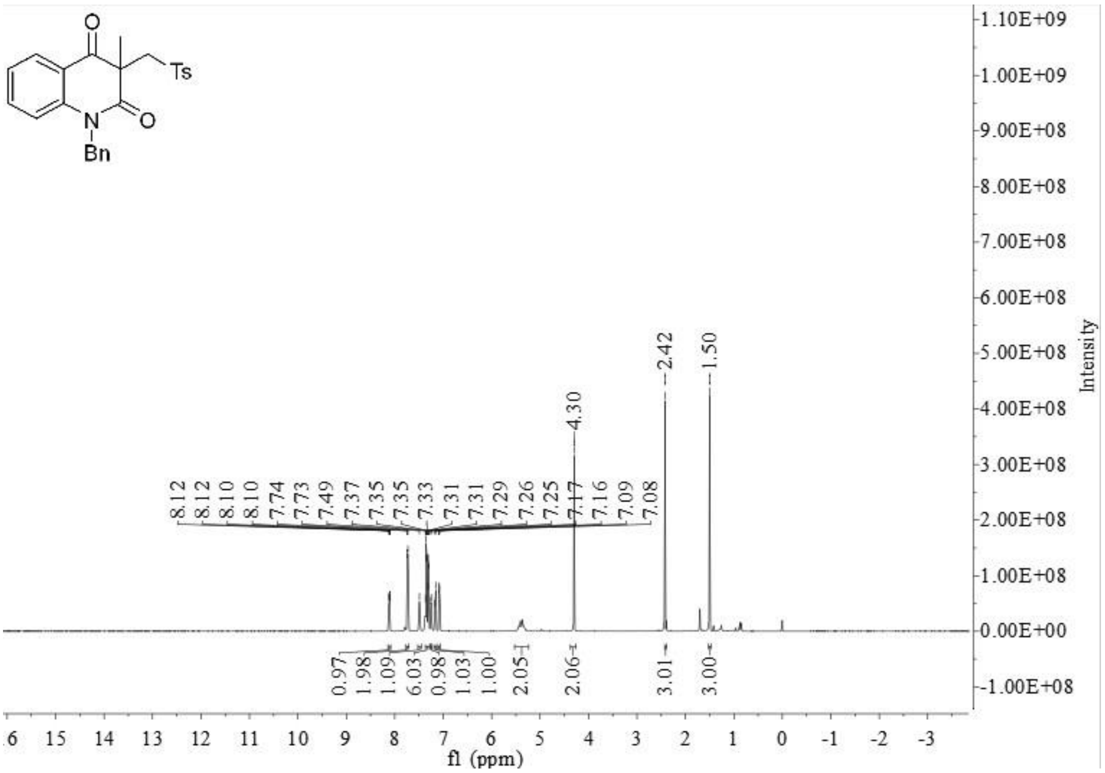
Ts
N
Bn
8.12
8.12
8.10
8.10
7.74
7.73
7.49
7.37
7.35
7.35
7.33
7.31
7.31
7.29
7.26
7.25
7.17
7.16
7.09
7.08
4.30
2.42
1.50
0.97
1.98
1.09
6.03
0.98
1.03
1.00
2.05
2.06
3.01
3.00
fl (ppm)
Intensity

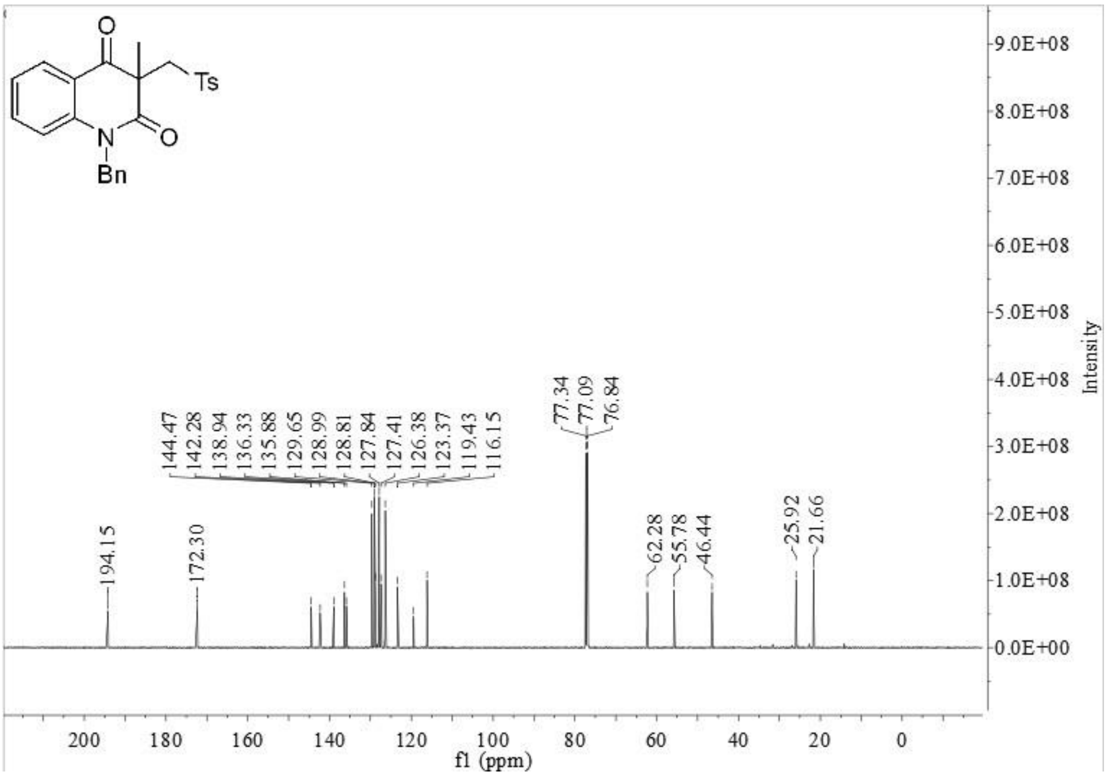
Ts
N
Bn
194.15
172.30
144.47
142.28
138.94
136.33
135.88
129.65
128.99
128.81
127.84
127.41
126.38
123.37
119.43
116.15
77.34
77.09
76.84
62.28
55.78
46.44
25.92
21.66
f1 (ppm)
Intensity

Compound 3t

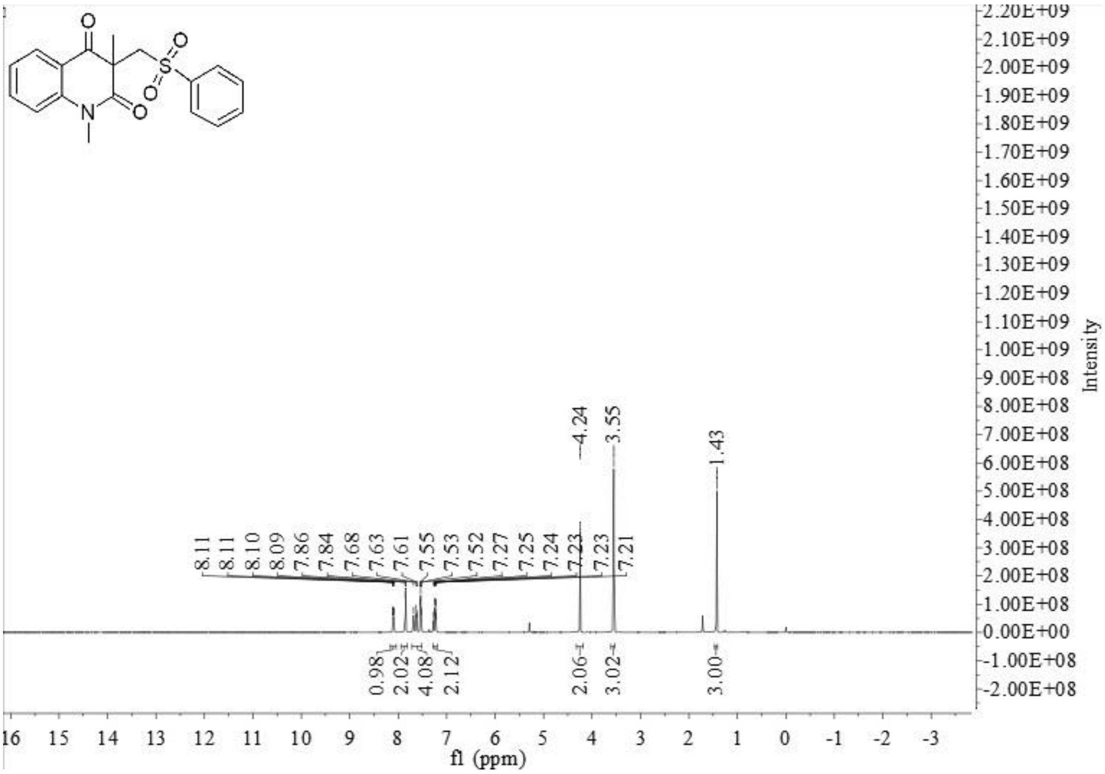
N
S
8.11
8.11
8.10
8.09
7.86
7.84
7.68
7.63
7.61
7.55
7.53
7.52
7.27
7.25
7.24
7.23
7.23
7.21
4.24
3.55
1.43
0.98
2.02
4.08
2.12
2.06
3.02
3.00
f1 (ppm)
Intensity

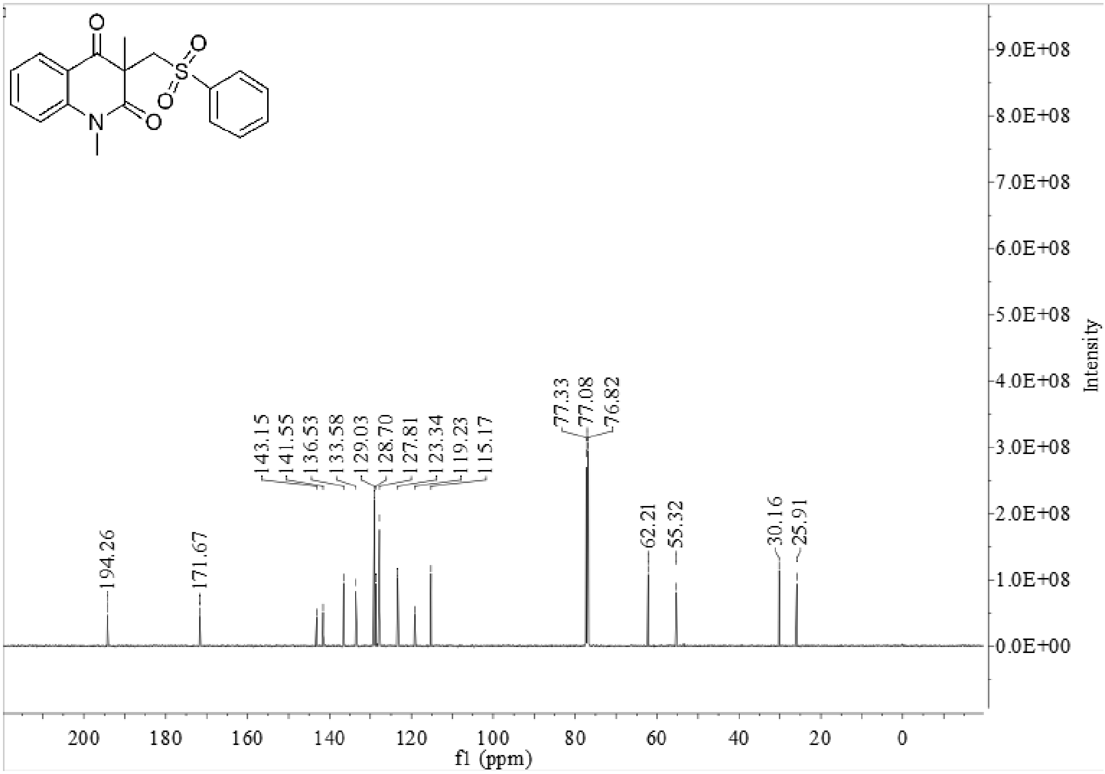
194.26
171.67
143.15
141.55
136.53
133.58
129.03
128.70
127.81
123.34
119.23
115.17
77.33
77.08
76.82
62.21
55.32
30.16
25.91
9.0E+08
8.0E+08
7.0E+08
6.0E+08
5.0E+08
4.0E+08
3.0E+08
2.0E+08
1.0E+08
0.0E+00
Intensity
200 180 160 140 120 100 80 60 40 20 0
f1 (ppm)

Compound 3u

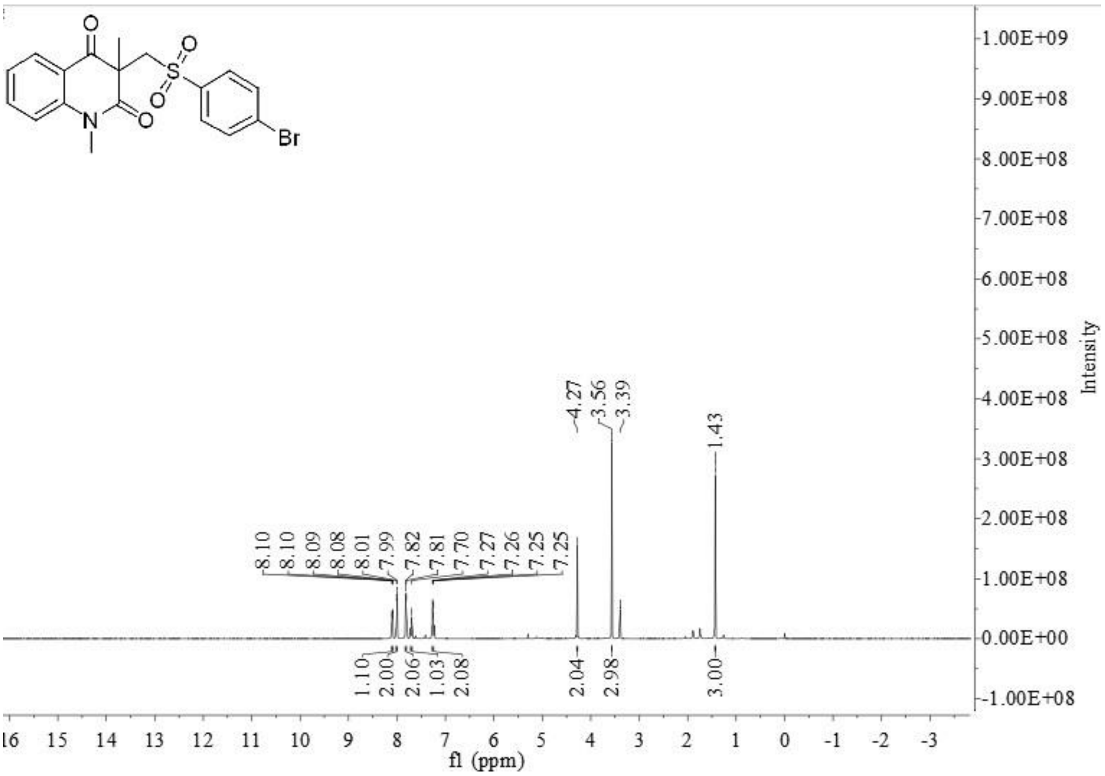
Br
8.10
8.10
8.09
8.08
8.01
7.99
7.82
7.81
7.70
7.27
7.26
7.25
7.25
4.27
3.56
3.39
1.43
1.10
2.00
2.06
1.03
2.08
2.04
2.98
3.00
1.00E+09
9.00E+08
8.00E+08
7.00E+08
6.00E+08
5.00E+08
4.00E+08
3.00E+08
2.00E+08
1.00E+08
0.00E+00
-1.00E+08
Intensity
16 15 14 13 12 11 10 9 8 7 6 5 4 3 2 1 0 -1 -2 -3
f1 (ppm)

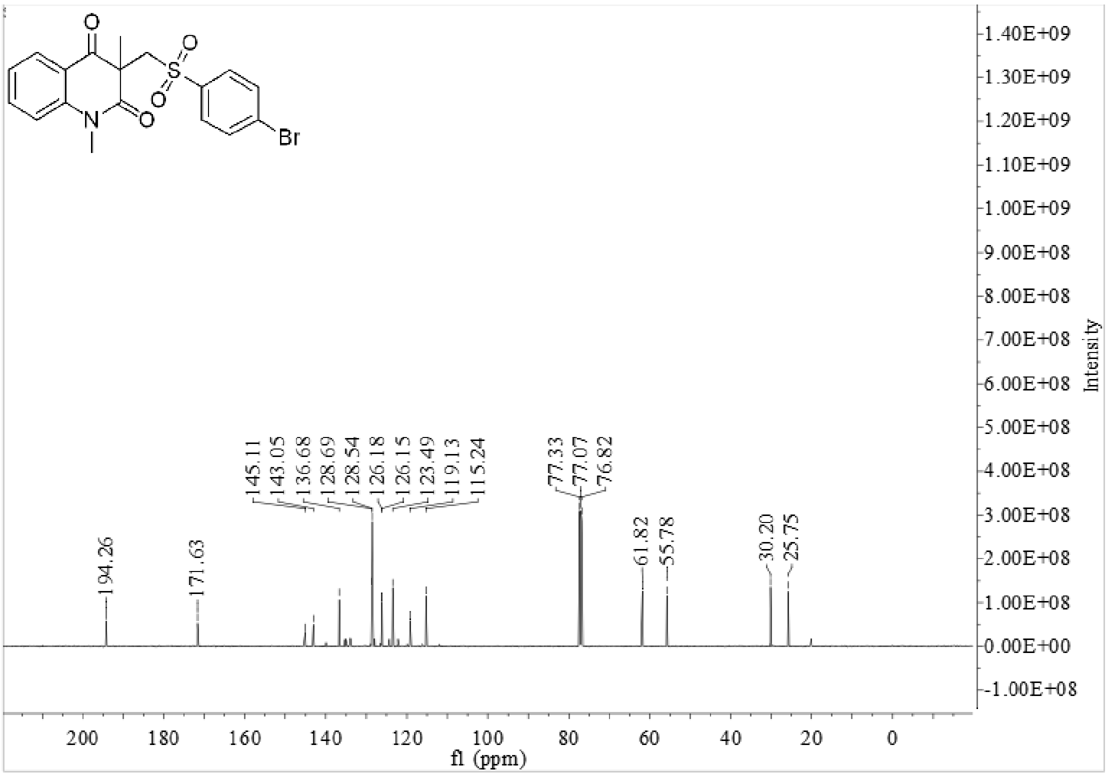

Br
194.26
171.63
145.11
143.05
136.68
128.69
128.54
126.18
126.15
123.49
119.13
115.24
77.33
77.07
76.82
61.82
55.78
30.20
25.75
fl (ppm)
Intensity

Compound 3v

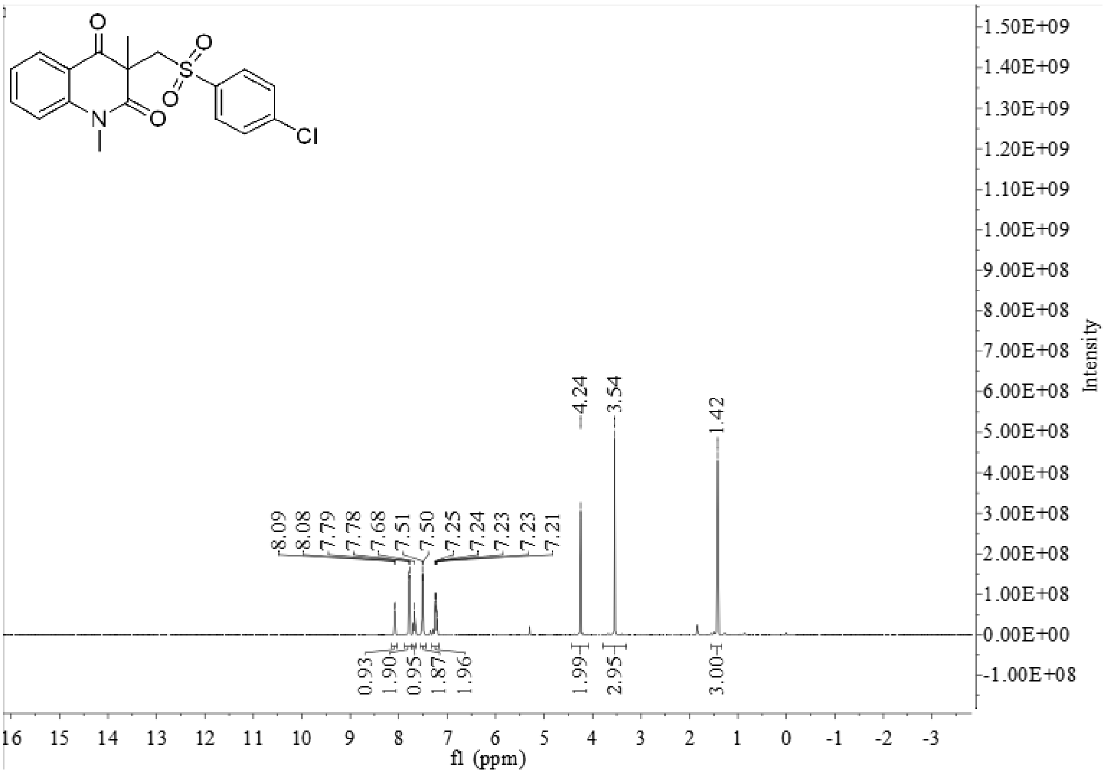

Cl
8.09
8.08
7.79
7.78
7.68
7.51
7.50
7.25
7.24
7.23
7.23
7.21
4.24
3.54
1.42
0.93
1.90
0.95
1.87
1.96
1.99
2.95
3.00
fl (ppm)
Intensity

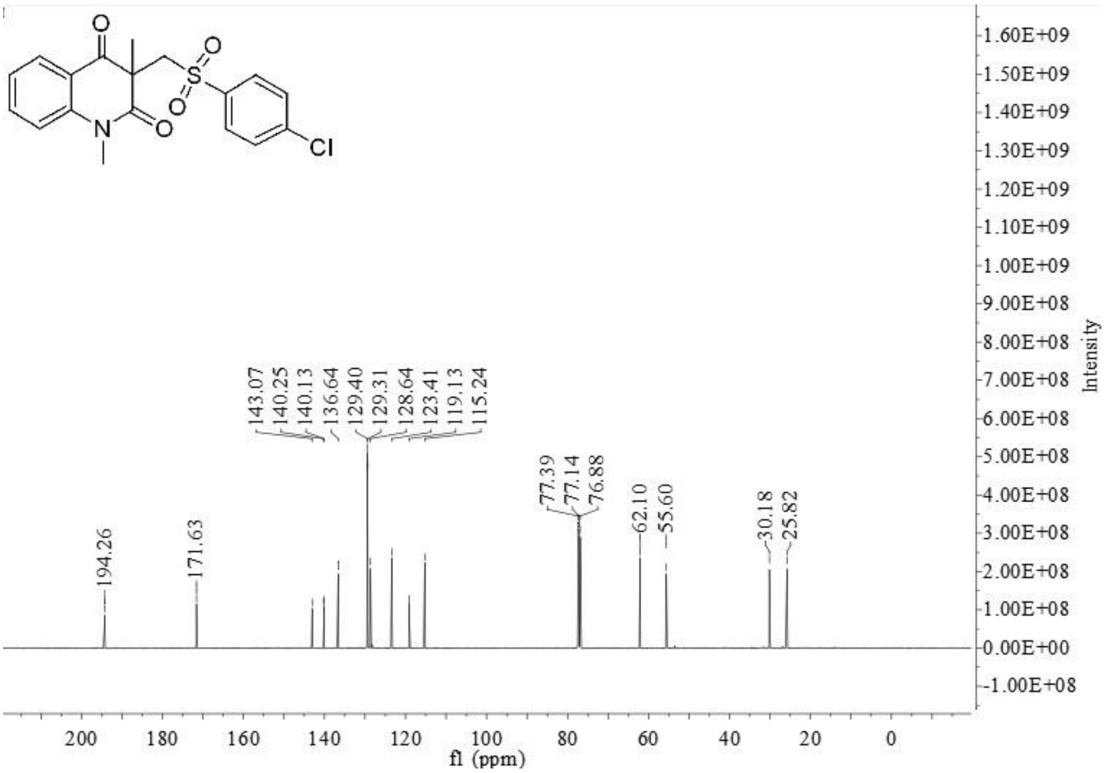

Compound 3w

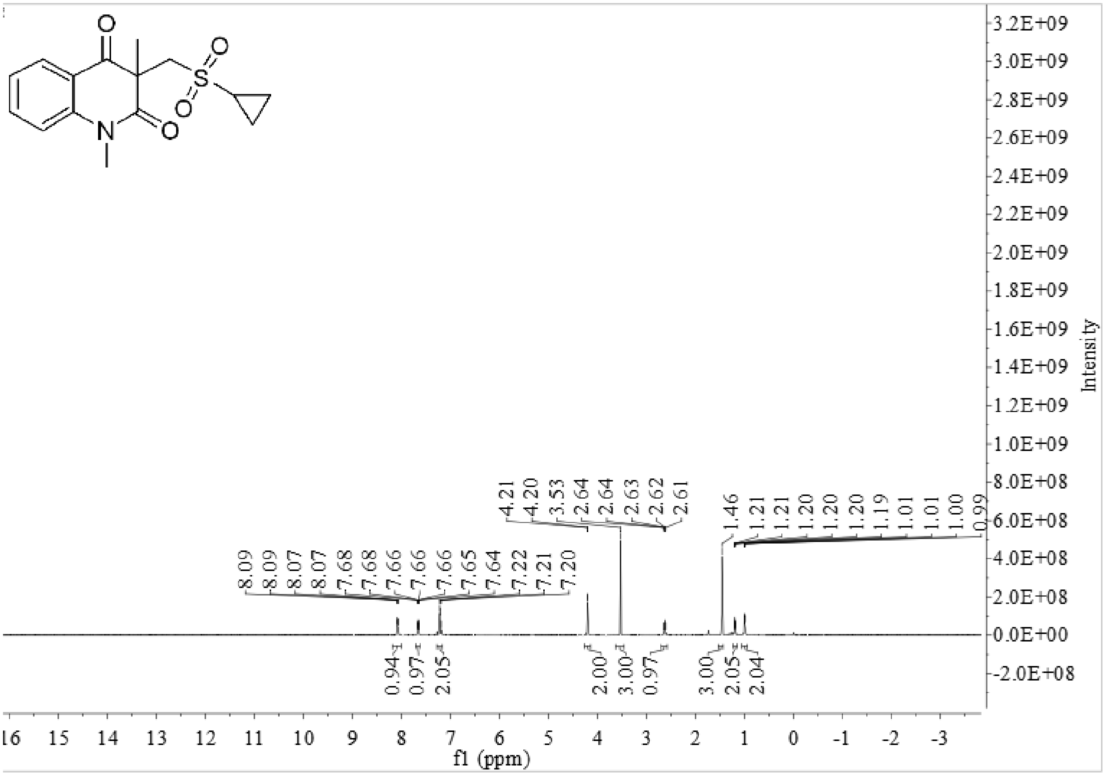

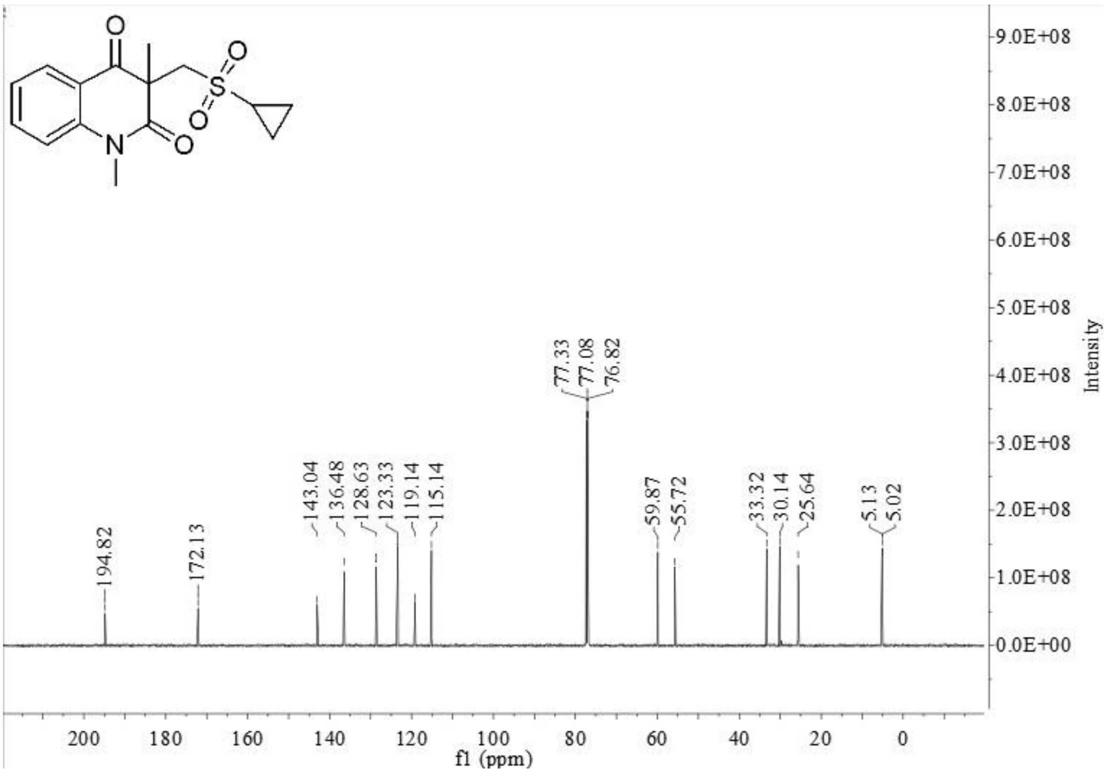

Compound 3x

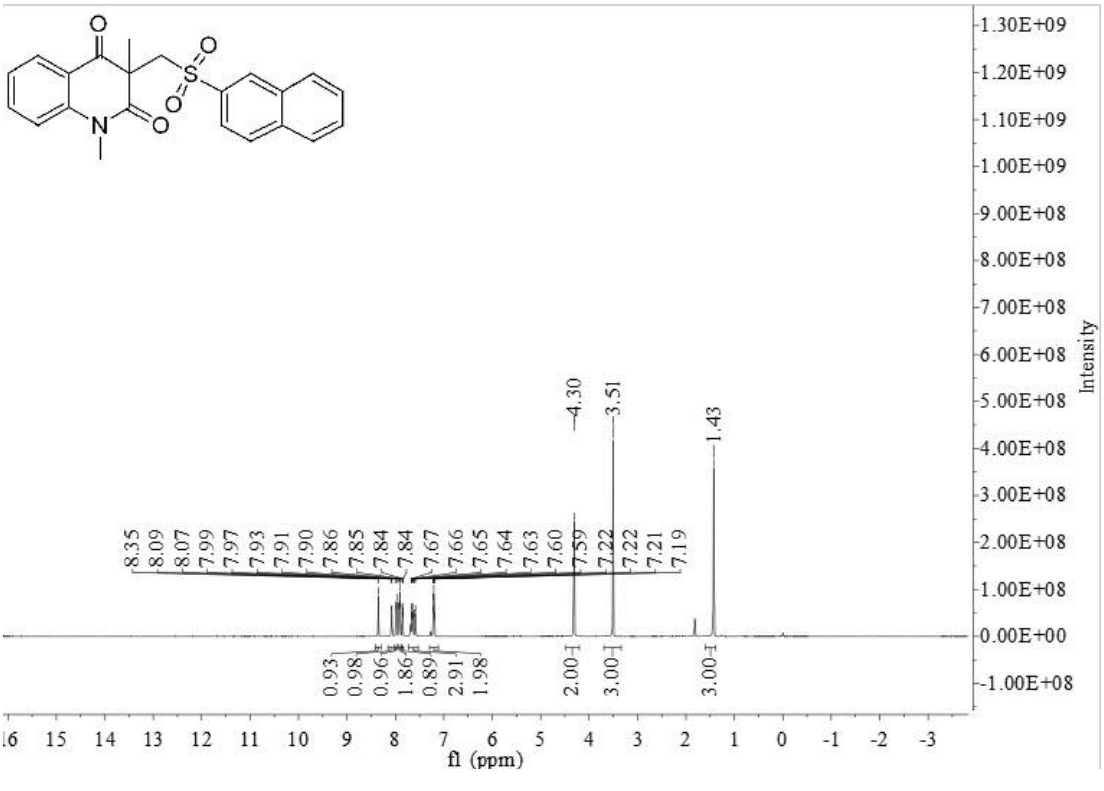

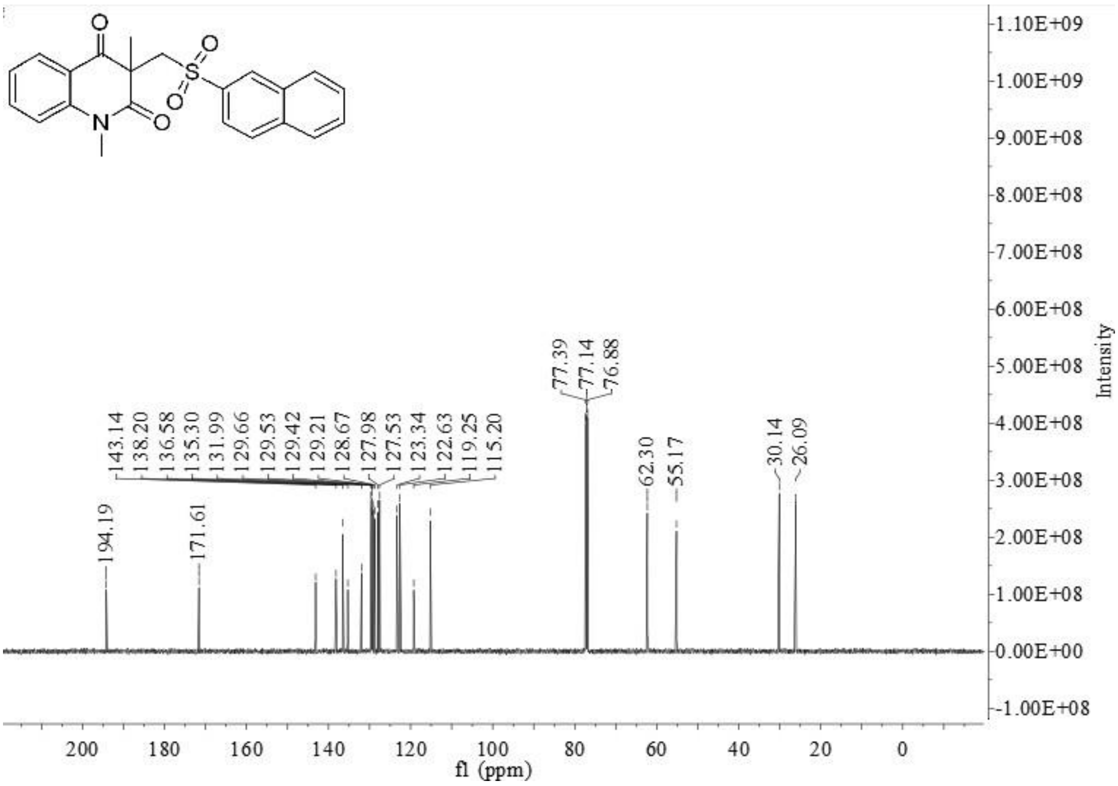

194.19
171.61
143.14
138.20
136.58
135.30
131.99
129.66
129.53
129.42
129.21
128.67
127.98
127.53
123.34
122.63
119.25
115.20
77.39
77.14
76.88
62.30
55.17
30.14
26.09
fl (ppm)
Intensity

Compound 3y

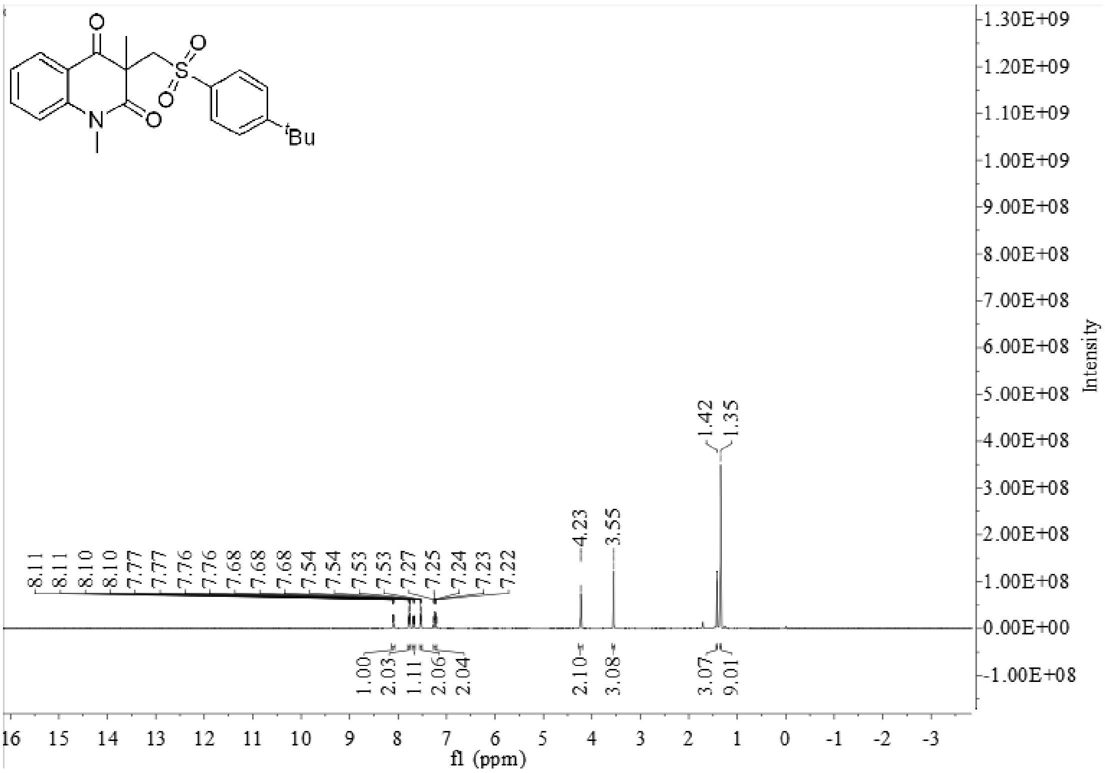

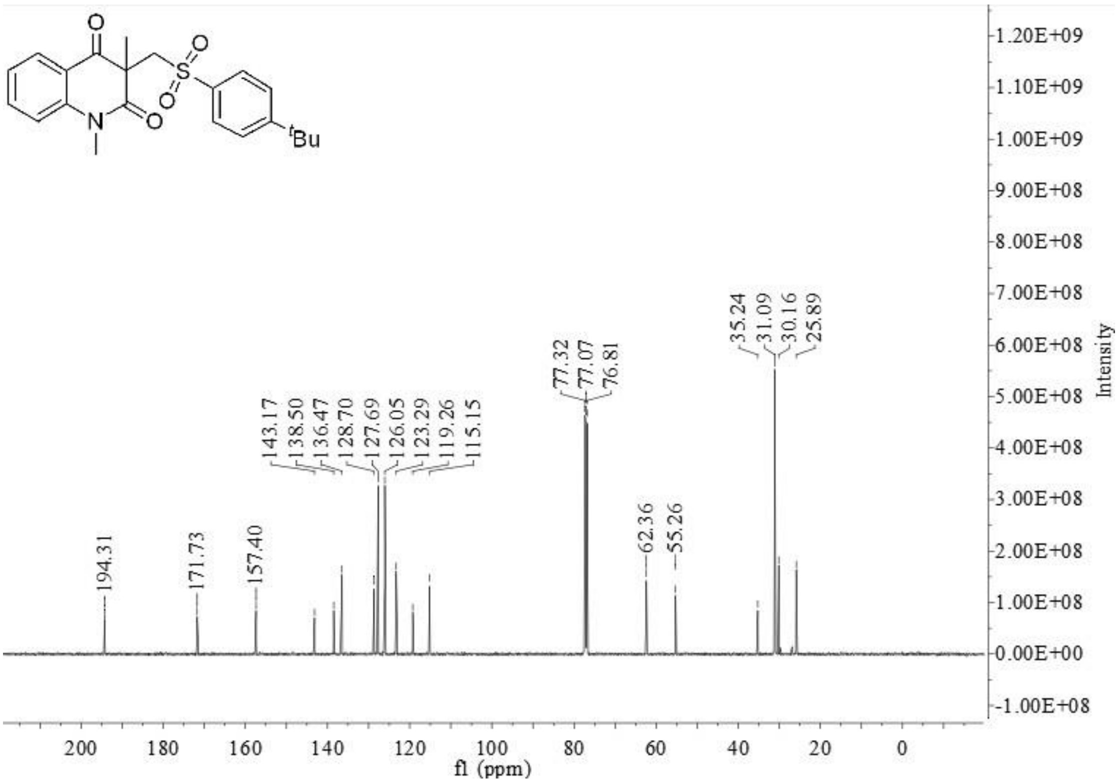
tBu
194.31
171.73
157.40
143.17
138.50
136.47
128.70
127.69
126.05
123.29
119.26
115.15
77.32
77.07
76.81
62.36
55.26
35.24
31.09
30.16
25.89
Intensity
fl (ppm)

Compound 3z

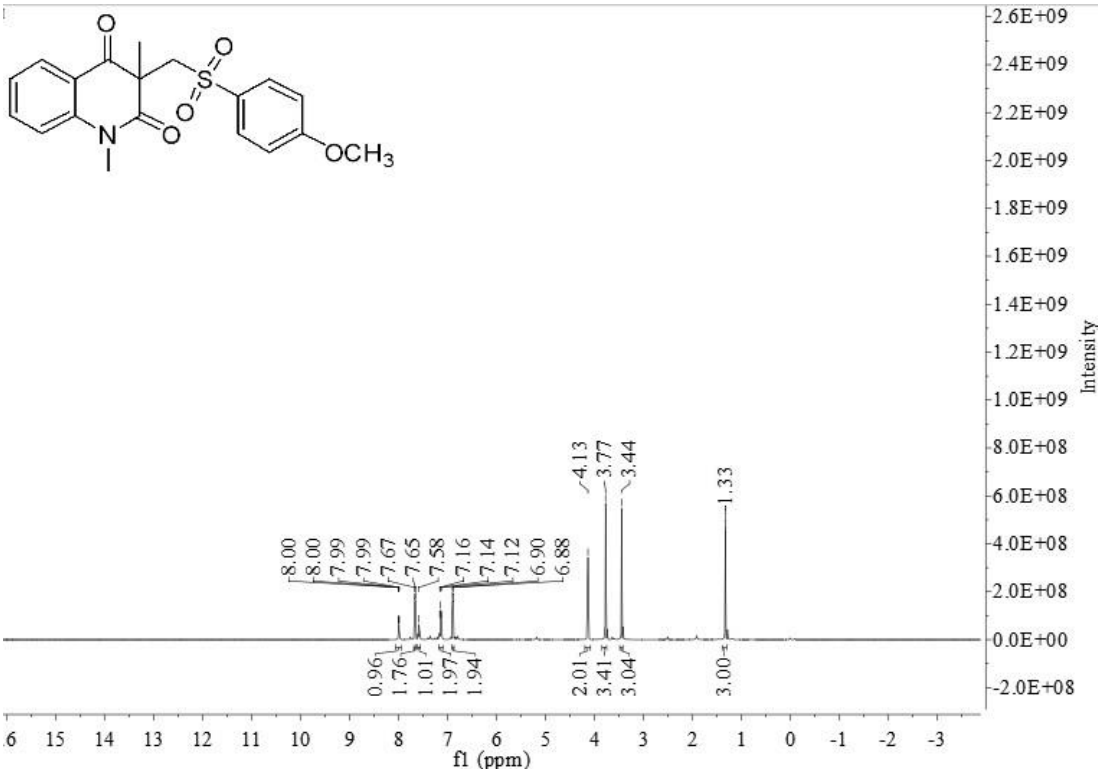
OCH3
8.00
8.00
7.99
7.99
7.67
7.65
7.58
7.16
7.14
7.12
6.90
6.88
4.13
3.77
3.44
1.33
0.96
1.76
1.01
1.97
1.94
2.01
3.41
3.04
3.00
Intensity
fl (ppm)

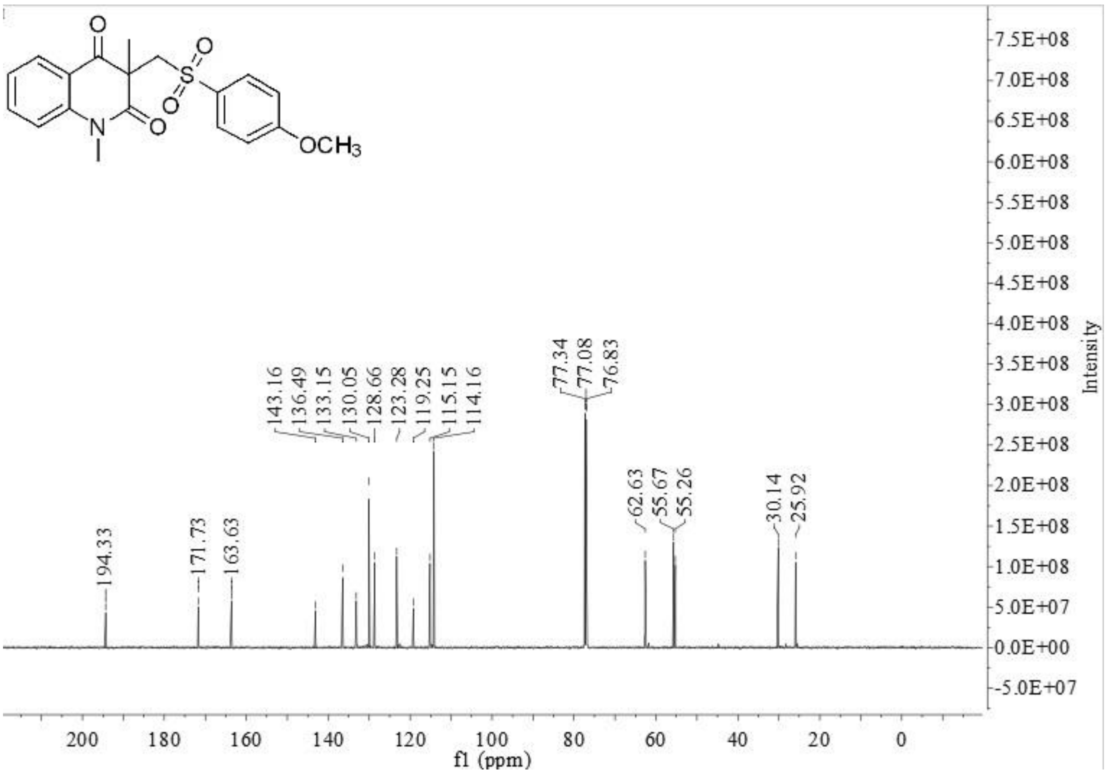

Compound 3aa

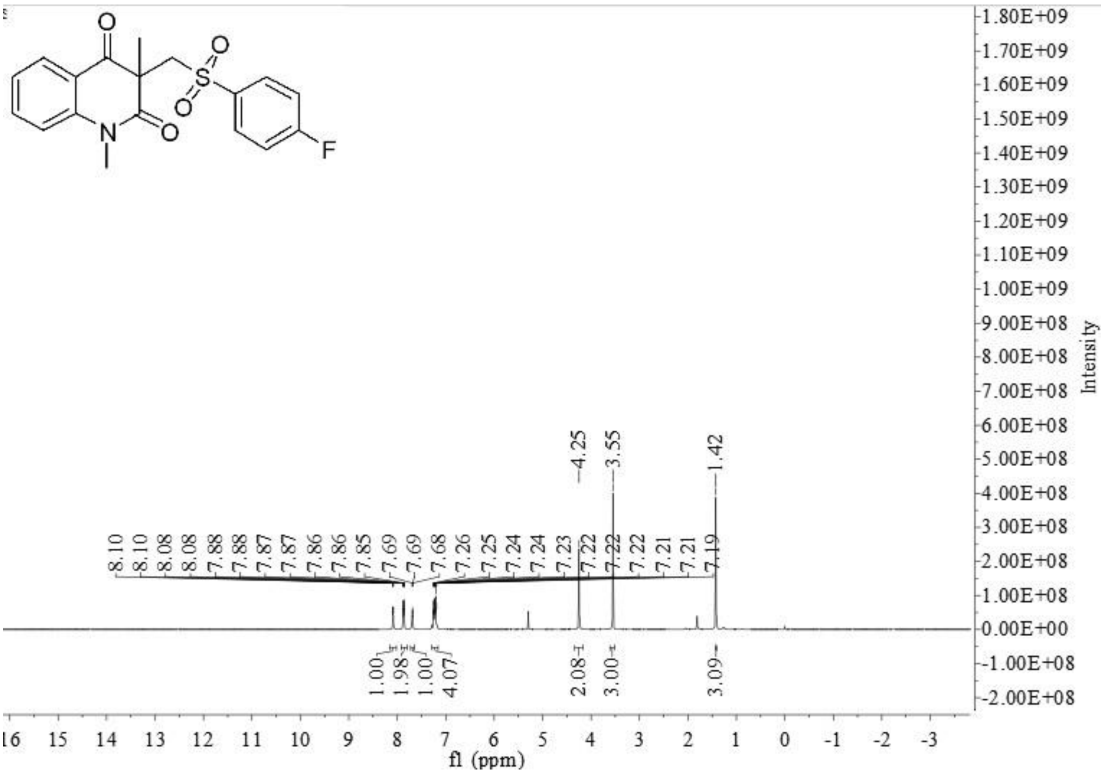

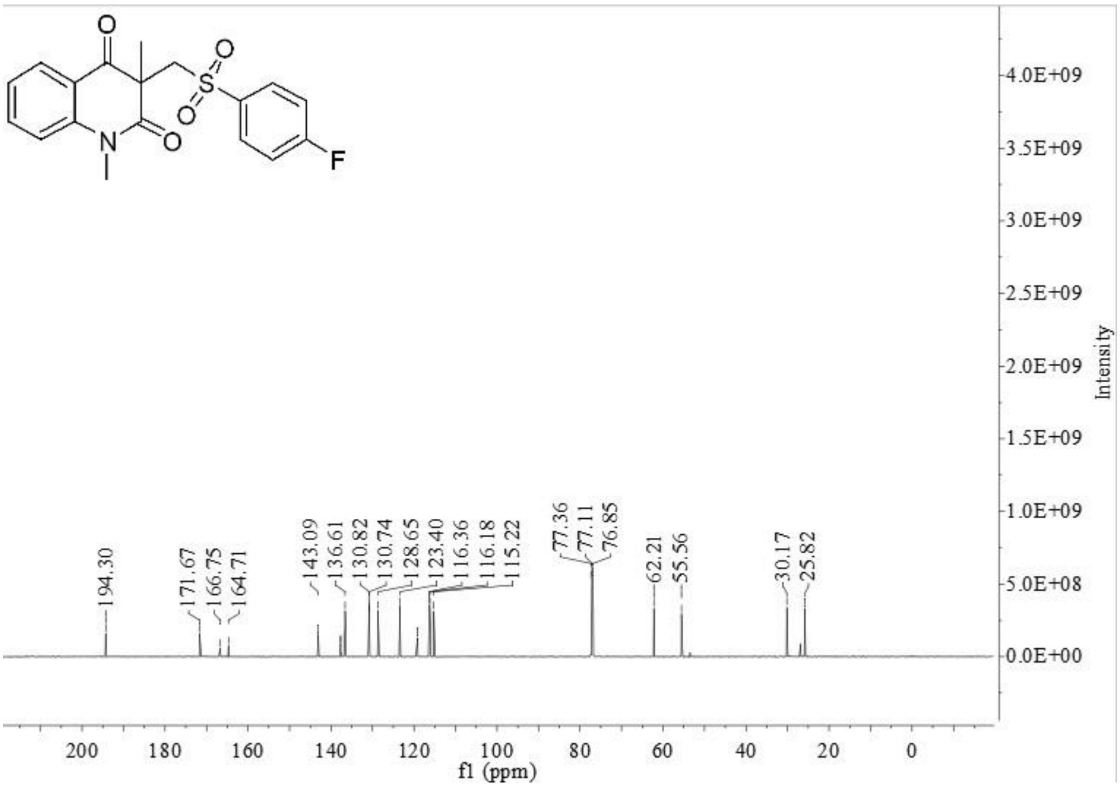
O
O
S
O
N
O
F
194.30
171.67
166.75
164.71
143.09
136.61
130.82
130.74
128.65
123.40
116.36
116.18
115.22
77.36
77.11
76.85
62.21
55.56
30.17
25.82
4.0E+09
3.5E+09
3.0E+09
2.5E+09
2.0E+09
1.5E+09
1.0E+09
5.0E+08
0.0E+00
Intensity
200 180 160 140 120 100 80 60 40 20 0
f1 (ppm)

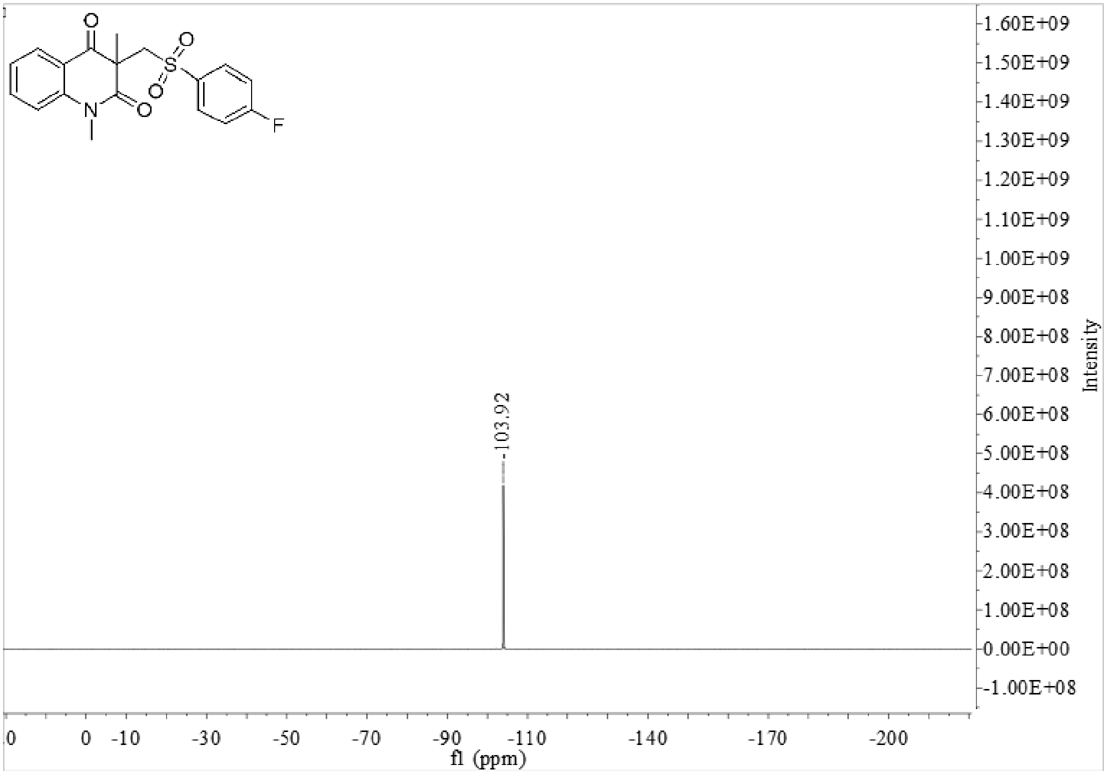
O
O
S
O
N
O
F
-103.92
1.60E+09
1.50E+09
1.40E+09
1.30E+09
1.20E+09
1.10E+09
1.00E+09
9.00E+08
8.00E+08
7.00E+08
6.00E+08
5.00E+08
4.00E+08
3.00E+08
2.00E+08
1.00E+08
0.00E+00
-1.00E+08
Intensity
0 0 -10 -30 -50 -70 -90 -110 -140 -170 -200
f1 (ppm)

Compound 4

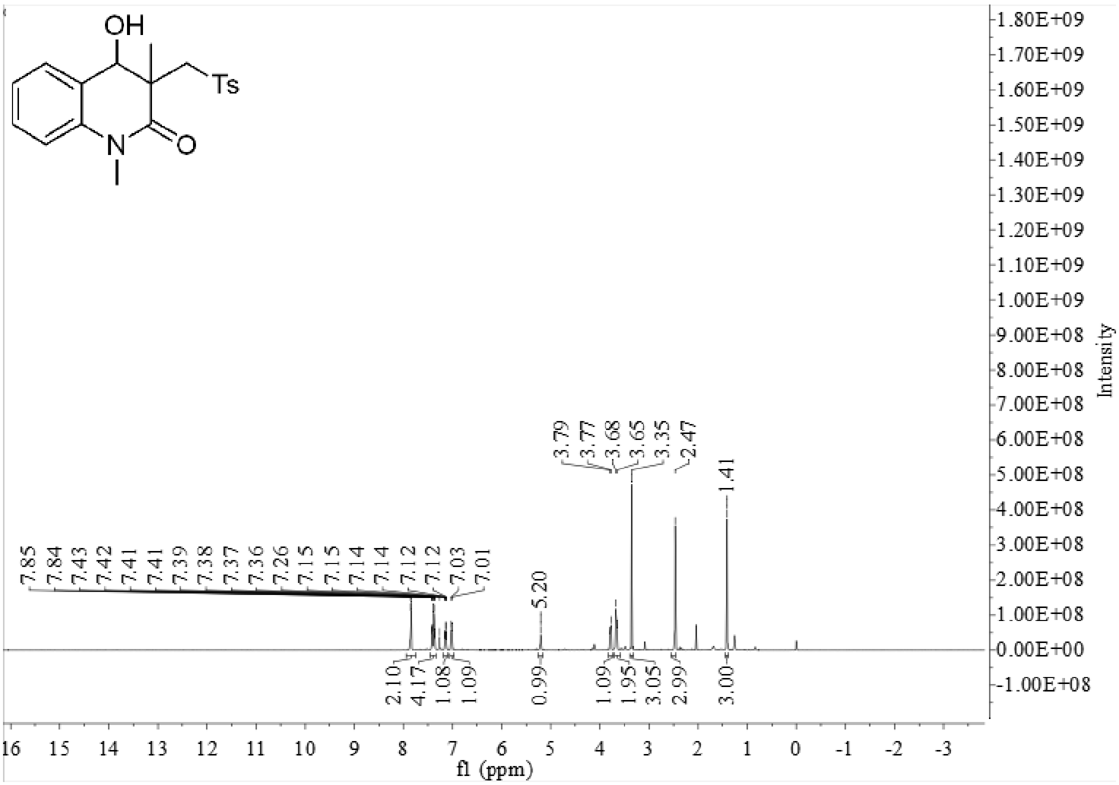

OH
Ts
N
O
7.85 7.84 7.43 7.42 7.41 7.41 7.39 7.38 7.37 7.36 7.26 7.15 7.15 7.14 7.14 7.12 7.12 7.03 7.01
5.20
3.79 3.77 3.68 3.65 3.35 2.47
1.41
2.10 4.17 1.08 1.09
0.99
1.09 1.95 3.05 2.99 3.00
fl (ppm)
Intensity

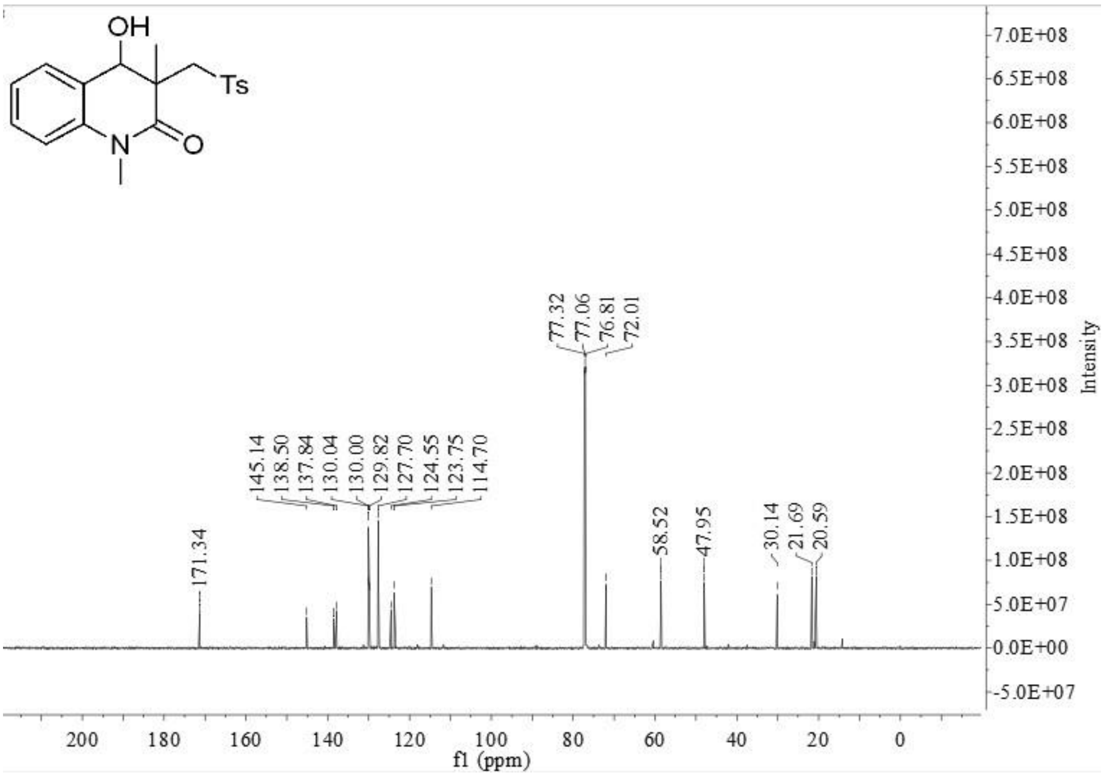

OH
Ts
N
O
171.34
145.14 138.50 137.84 130.04 130.00 129.82 127.70 124.55 123.75 114.70
77.32 77.06 76.81 72.01
58.52
47.95
30.14 21.69 20.59
fl (ppm)
Intensity

Compound 5

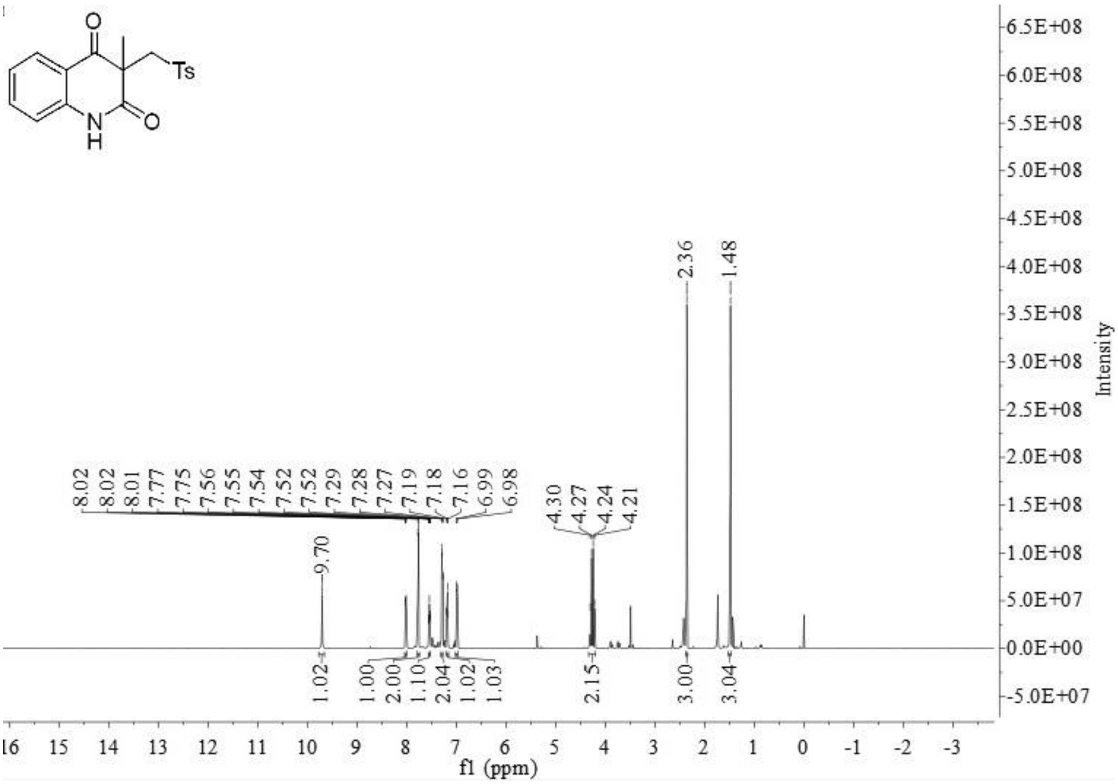
Ts
NH
O
8.02
8.02
8.01
7.77
7.75
7.56
7.55
7.54
7.52
7.52
7.29
7.28
7.27
7.19
7.18
7.16
6.99
6.98
9.70
4.30
4.27
4.24
4.21
2.36
1.48
1.02
1.00
2.00
1.10
2.04
1.02
1.03
2.15
3.00
3.04
f1 (ppm)
Intensity

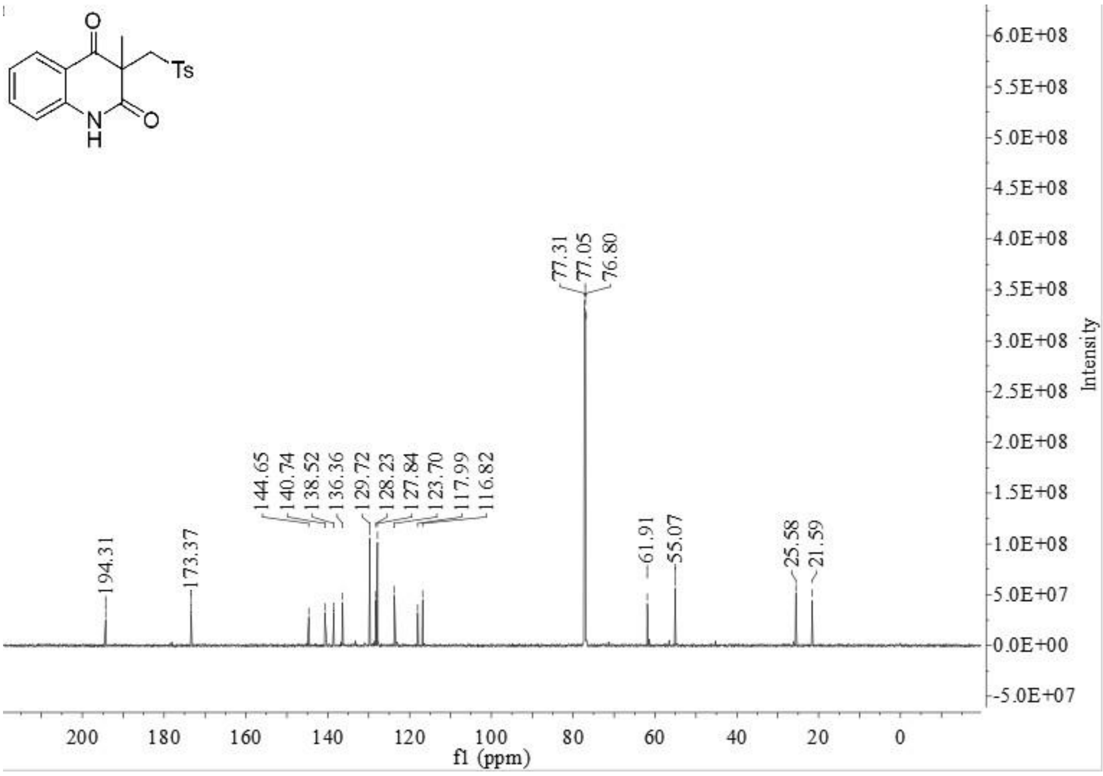
Ts
NH
O
194.31
173.37
144.65
140.74
138.52
136.36
129.72
128.23
127.84
123.70
117.99
116.82
77.31
77.05
76.80
61.91
55.07
25.58
21.59
f1 (ppm)
Intensity
